# Predicting the growth of asymptomatic small abdominal aortic aneurysms (AAA) based on deep learning

**DOI:** 10.3389/fphys.2025.1704428

**Published:** 2026-01-27

**Authors:** Jiaxin Cheng, Zhiqiang Zhang, Yasong Wang, Yu Sun, Nan Wang, Xiaozeng Wang, Sihan Wang

**Affiliations:** 1 College of Medicine and Biological Information Engineering, Northeastern University, Shenyang, Liaoning, China; 2 National Key Laboratory of Frigid Zone Cardiovascular Disease, Cardiovascular Research Institute and Department of Cardiology, General Hospital of Northern Theater Command, Shenyang, Liaoning, China; 3 Department of Radiology, General Hospital of Northern Theater Command, Shenyang, Liaoning, China; 4 State Key Laboratory of Robotics, Shenyang Institute of Automation, Chinese Academy of Sciences, Shenyang, Liaoning, China

**Keywords:** multi-head self-attention, computed tomography angiography, growth prediction, deep learning, abdominal aortic aneurysm

## Abstract

Accurate prediction of asymptomatic small abdominal aortic aneurysm (AAA) growth is crucial for risk stratification and personalized surveillance. This study developed an end-to-end deep learning framework to predict rapid expansion (≥0.5 cm/6 months) using computed tomography angiography (CTA) images from 81 asymptomatic patients with small AAA (30 rapid-growth and 51 stable patients). The pipeline integrated three core components: a ResNet50 classifier for identifying aortic images (99.86% accuracy, 99.91% F1-score), a YOLOv11 detector for localizing aneurysms (precision–recall: 0.902), and a MedMamba-based feature fusion model that combined imaging features with clinical metadata via multi-head self-attention. Model robustness was ensured through stratified 5-fold cross-validation and comprehensive data augmentation. The fusion model achieved a predictive accuracy of 98.75% and an F1-score of 97.78, outperforming seven classical deep learning backbones. Furthermore, explainability analyses confirmed the model’s reliance on established clinical risk factors and highlighted biologically plausible imaging regions for prediction. The proposed ResNet50–YOLOv11–MedMamba framework demonstrates the feasibility of automating AAA growth prediction directly from CTA and shows promising potential to enhance clinical decision-making.

## Introduction

Abdominal aortic aneurysm (AAA) constitutes a pathological dilation of the infrarenal abdominal aorta and is often subclinical until the time of acute rupture, a complication with prehospital mortality exceeding 80% (Schanzer et al., 2021; Isselbacher et al., 2022). Current clinical management relies on serial diameter monitoring, with intervention typically recommended when the maximum diameter exceeds 50 mm for men or a lower threshold for women (Wanhainen et al., 2024). The diameter is the most commonly used risk marker in AAA disease. It is manually measured clinically by ultrasound or multiplanar reconstruction of computed tomography angiography (CTA) perpendicular to the centerline. Current predictions of the AAA rupture risk, and consequently the indications for preventive treatment, are based on the maximum anterior-posterior diameter, measured perpendicular to the centerline with three-dimensional reconstructed CTA images, and growth rate. However, this size-only paradigm is an imperfect predictor of risk. A significant proportion of ruptures occur in aneurysms below this surgical threshold (Collaborators, 2013; Spanos et al., 2020), with large cohort studies reporting an annual rupture risk of 0.03% for small AAAs (Oliver-Williams et al., 2019). This critical limitation underscores the urgent need for better predictors of aneurysm behavior. Consequently, accurately forecasting the AAA growth rate has become a central research priority, as it is crucial for surgical planning and personalized surveillance, with guidelines recommending intervention when growth exceeds 0.5 cm per 6 months (Ullery et al., 2018; Isselbacher et al., 2022).

The pursuit of improved prediction has incorporated biological variables (e.g., C-reactive protein and D-dimers) and morphological parameters from CTA (Zhu et al., 2020; Cersit et al., 2021; Fernandez-Alonso et al., 2022; Kontopodis et al., 2022; Ristl et al., 2023; Vanmaele et al., 2025). Furthermore, the pathobiology of the perivascular environment is now recognized as a key contributor to AAA progression (Wang et al., 2022; Zhang et al., 2023; Lv et al., 2024). Deep learning offers a powerful approach to automatically extract prognostic features from images. Previous efforts have largely relied on radiomics derived from manual segmentations or reconstructed geometries for hemodynamic modeling. While valuable, this dependence on manual annotation introduces observer variability, limits scalability, and confines models to a predefined set of human-engineered features, potentially overlooking subtler prognostic patterns in the raw data (Kim et al., 2023; Wang et al., 2023).

To overcome these limitations, we propose a novel, end-to-end deep learning framework that automates the entire pipeline from raw CTA images to growth prediction. Our framework, ResNet50–YOLOv11–MedMamba, is designed to eliminate manual intervention through a three-stage cascade: it first identifies and filters relevant aortic images, then precisely localizes the aneurysm region, and finally fuses the automatically extracted imaging features with clinical metadata for a holistic assessment. This study aims to validate whether this fully automated approach can accurately predict rapid expansion (≥0.5 cm/6 months) of small AAAs, thereby offering a scalable, potentially more robust alternative to existing methods.

## Methods

**FIGURE 1:**
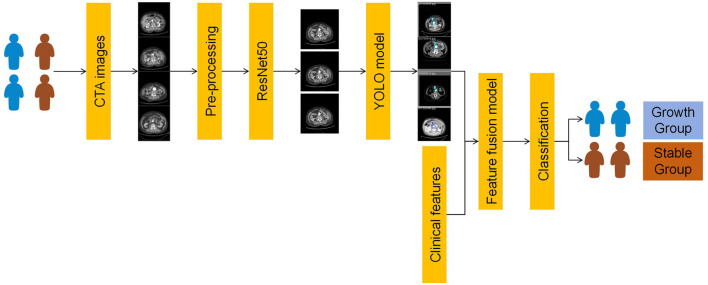
Framework of our prediction of AAA growth. AAA, abdominal aortic aneurysm; YOLO, You Only Look Once.

This section provides an overview of the methods employed in this study, with a particular focus on the feature fusion strategy and the application of the ResNet50, YOLOv11, and MedMamba models. We have detailed the data collection process, CTA data acquisition, definition, and the performance metrics used to evaluate our method. By integrating clinical data and CTA image feature-extraction techniques, we aim to improve the accuracy of predicting AAA growth in asymptomatic patients. Each subsection provides a comprehensive overview of the process used, ensuring the clarity of our research methods and facilitating the reproducibility of this work. The framework of the proposed algorithm for our prediction of AAA growth is shown in Figure 1.

### Data collection

This retrospective study consecutively screened 289 patients with small AAAs who were treated in our center from January 2019 to December 2023. Among them, 135 patients who underwent intervention due to symptoms were excluded, 61 patients who did not undergo imaging follow-up were excluded, 10 patients were lost to follow-up, and two patients died. Finally, 81 patients were included in this study. According to whether the growth rate was >0.5 cm/6 months, they were divided into the rapid-growth group (n = 30) and the stable group (n = 51). The CTA images of these patients were obtained from the institutional Picture Archiving and Communication Systems at our center for the study, and the patients’ clinical data were obtained from the electronic medical record system. This study followed the ethical guidelines of the Helsinki Declaration and was approved by the ethics committee. The Ethics Committee of General Hospital of Northern Theater Command approved this study with an Ethics Batch Number Y (2024)356. Informed consent was waived due to the retrospective design and the use of fully anonymized, de-identified data.

### CTA data acquisition

CTA examinations were conducted via a 256-slice multidetector computed tomography system (Brilliance iCT, Philips Healthcare). The scanning protocol included the following technical specifications: detector collimation of 128 mm × 0.625 mm, a gantry rotation time of 270 ms, automated tube voltage selection ranging from 100 kVp to 120 kVp based on body mass index, and tube current modulation between 500 mAs and 700 mAs. Image acquisition utilized retrospective electrocardiogram gating with intravenous administration of iodinated contrast medium (ioversol 320 mgI/mL) at weight-adjusted doses of 1–1.5 mL/kg and injection rates of 4–6 mL/s. CTA images were reconstructed at a window centered at the peak aortic enhancement phase, which can be determined by a test bolus or bolus-tracking technique. For most patients, this phase may occur at approximately 20–30 s after the start of contrast injection, but it can vary depending on the patient’s cardiovascular status and the injection parameters. CTA images were reconstructed at a window centered at 75% of the R‒R interval, with a section thickness of 0.625 mm and a reconstruction increment of 0.5 mm.

### Definition

A small AAA is defined as the diameter of the abdominal aorta between 3 cm and 5 cm in women and between 3 cm and 5.5 cm in men (Isselbacher et al., 2022), and the maximum diameter was measured by two radiologists based on multiplanar reconstruction. The rapid growth of AAA is defined as an increase of more than 5 mm every 6 months (Ullery et al., 2018). Smoking history was defined as an individual’s lifetime consumption of 100 or more cigarettes (Rigotti et al., 2024), and drinking history was defined as daily consumption of at least 50 mL of liquor, at least once a week, for a duration of at least 6 months, including current drinkers and those who stopped drinking but met the aforementioned criteria (Herzog et al., 2024).

### Model framework strategy

The CTA data collection adopts the RGB image format, with variable anatomical semantics. To ensure consistent model input, we used a classifier based on ResNet50 pre-trained on ImageNet to automatically identify CTA images containing the abdominal aorta. 182,291 CTA slices were uniformly preprocessed to a resolution of 224 × 224. The ImageNet standard normalization was applied (mean = [0.485, 0.456, 0.406], standard deviation = [0.229, 0.224, 0.225]), and the center cropping strategy was adopted to maintain the integrity of the anatomical structure. This model has undergone over 100 training epochs using a batch size of 64 and an initial learning rate of 0.001, and the Adam optimizer was employed with step-decayed learning rate scheduling (multiplied by 0.1 every 50 epochs), enabling it to reliably perform anatomical filtering for image selection. The loss function used is the cross-entropy loss.

The CTA images that contain the abdominal aorta were labeled by LabelMe and used as coordinates for feature extraction by the You Only Look Once (YOLO) models (Aly et al., 2021; Hechkel and Helali, 2025), located in the local area of the abdominal aorta, with labels set as normal aorta and AAA images. The local abdominal aorta features extracted by YOLO were used for the feature fusion. A comparative analysis framework was implemented to evaluate four YOLO variants (v5, v8, v10, and v11) under identical experimental conditions. Each model was trained for 100 epochs with a batch size of 16 and an input resolution of 640 pixels × 640 pixels. The optimizer is Stochastic Gradient Descent (SGD) with Nesterov momentum (μ = 0.937). The initialized learning rate was set at 0.01 with fixed scheduling and L2 regularization weight decay (λ = 5 × 10^4^). Detection performance was assessed using mean average precision (mAP) at an intersection over union (IoU) = 0.5 threshold (mAP@50) and mAP averaged over IoU thresholds 0.5 to 0.95 (mAP@50–95). Computational efficiency was measured by total training time.

**FIGURE 2:**
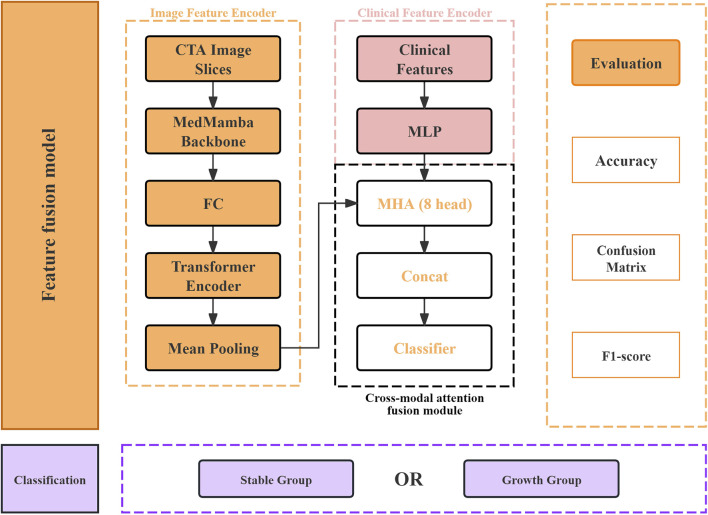
Overall framework of the feature fusion model. CTA, computed tomography angiography; FC, fully connected; MLP, multilayer perceptron; MHA, multi-head self-attention.

We propose a novel multimodal model that integrates CTA image slices and clinical features via a feature fusion mechanism, as shown in Figure 2. The architecture adopts a dual-pipeline architecture and comprises three innovative components: an image feature encoder, a clinical feature encoder, and a cross-modal attention fusion module. The dataset was randomly partitioned into training, validation, and test sets at an 8:1:1 ratio at the patient level to ensure independence. For the image feature encoder, the MedMamba backbone (Yue and Li, 2024) was selected for its specific design to model long-range dependencies in medical image sequences, a capability highly relevant to our task. The output of MedMamba from all slices was then treated as a sequence and passed into a transformer encoder, which was fed into the image encoder backbone to extract slice-level feature representations. The resulting features were then projected into a shared 512-dimensional latent space via a projection head consisting of linear layers, batch normalization, and dropout. Mean pooling is applied to produce a fixed-length image embedding for each patient.

**FIGURE 3:**
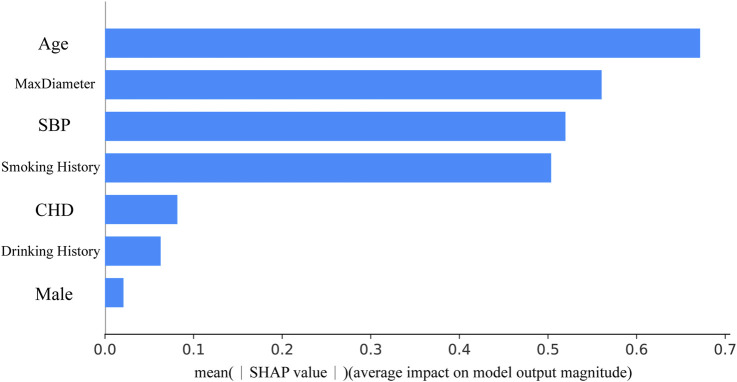
Feature importance ranking obtained through feature selection based on extreme gradient boosting. MaxDiameter, maximum diameter of abdominal aortic aneurysm; SBP, systolic blood pressure; CHD, coronary atherosclerotic heart disease.

To prevent data leakage, all preprocessing steps were performed independently on each partition. Clinical features with missing values exceeding 50% were excluded from the analysis. For the remaining missing data, four imputation methods were evaluated: k-nearest neighbors (KNN), median imputation, random forest imputation, and multiple imputation (Lee and Styczynski, 2018). KNN imputation (k = 5) was selected because it best preserved the original data distribution and was subsequently applied to each subset (training, validation, test) separately. Continuous features were standardized to a mean of zero and a unit variance using the StandardScaler method. The categorical outcome variable (intact vs. ruptured) was binarized as 0 and 1, respectively. Prior to feature selection, multicollinearity was addressed by identifying and eliminating highly correlated features (Pearson’s |r| > 0.8). We implemented extreme gradient boosting (XGBoost) to screen clinical features. The top seven most discriminative features from each method were selected to form candidate feature subsets. The Shapley Additive exPlanations (SHAP) value is shown in Figure 3.

The clinical features were analyzed via a two-layer multilayer perceptron (MLP), with intermediate batch normalization and dropout layers, to improve generalizability. The final output is mapped into the same latent space as the image embedding (i.e., 1,024 dimensions), enabling subsequent multimodal fusion. A key innovation of our architecture lies in the cross-modal attention fusion module. Here, we treat the image-derived and clinical-derived embeddings as two tokens and input them into a multi-head self-attention (MHA) block. This allows the model to explicitly learn modality-aware representations by attending to both intra- and intermodal interactions. The output vectors from each modality are then concatenated and passed through a multilayer classifier for binary classification. The thickness of the image layer is 5 mm, and the 2D images are all 224×224. All images are preprocessed using the low threshold zero processing method, with the threshold experimentally set at 200. During the training process, the Adam optimizer is used. In the training process, the 5-fold cross-validation method with category balance weights is used, and robustness and adaptive learning rate adjustment (initial 1e−4, reduced by 10% on the platform) are implemented. The loss function is also the cross-entropy loss. The batch size is set to 4, adjusted according to memory requirements for comparing models. Through normalization of shear (max = 1.0), supplementation, and maintaining gradient stability through strict CTA data augmentation, including random planar rotation (±15°), horizontal flipping (50%), ±20% brightness, contrast, and saturation, it is made applicable to medical data. To ensure the reduction of overfitting and underfitting of the model, the early stopping strategy is a common method to prevent model overfitting, with patience = 10. We adopted a stratified 5-fold cross-validation for dataset division to ensure that each fold maintains the original class distribution ratio. To address the class imbalance problem (with a class ratio of 30:51), we used the weighted cross-entropy loss function based on class frequency.

### Implementation configurations

Our architecture is implemented on PyTorch 2.7.0, and experiments are conducted on a workstation equipped with an NVIDIA GeForce RTX 4090 GPU and providing up to 24 GB of VRAM, NVIDIA driver version 576.52, and CUDA 12.8.

### Comparative experiment

In the comparative experiment, we set up three types of experiments. The first one is the comparison between the feature-extraction module MedMamba and the classical models, including VGG16, ResNet18, ResNet50, ResNet101, DenseNet121, ViT-B/16, and MedViT. The second one is comparing the results of feature fusion achieved by combining other attention mechanisms with the MedMamba model, including the convolutional block attention module (CBAM), channel attention, cross attention, the squeeze-and-excitation (SE) module, and the global attention mechanism (GAM). Additionally, we explored the number of heads in the MHA mechanism and compared the results of 4 heads, 8 heads, 16 heads, and 32 heads, respectively.

### Model interpretability and visualization

To interpret the predictions of our trained model, we employed gradient-weighted class activation mapping (Grad-CAM) to generate visual explanations of its decision-making process (Wang et al., 2025; Zhao et al., 2025). This technique produces coarse localization heatmaps by leveraging gradient information flowing into the final convolutional layer of the network, highlighting regions of the input image that are most influential for predicting a specific class while preserving spatial information. A major advantage of Grad-CAM is that it requires no modifications to the model architecture and involves no additional training. In this study, we applied Grad-CAM to the final convolutional layer to visualize activation patterns corresponding to the model’s prediction of the AAA growth.

### Statistical analysis

To evaluate the classification results of the deep learning models for the overall group, we used sensitivity, specificity, accuracy, F1-score, precision, the mAP@50, and the area under the curve (AUC). The evaluation formula is available in the supplementary materials. The statistical analyses were performed using IBM® SPSS® version 26 (IBM, Armonk, NY) and Python 3.12.9.

## Results

### Baseline characteristics

Overall, we evaluated 81 patients with asymptomatic and image-monitored small AAA without intervention. Most were men (90.12%), with a median age of 68 years. Among them, 30 patients met the criteria for rapid growth (0.5 cm/6 months), and the other 51 patients were of the stable type. The proportion of smoking history among the patients in the rapid-growth group was significantly higher than that of the patients in the stable group (P = 0.016). No statistically significant differences in other baseline characteristics were found between the two groups (all P > 0.05). Detailed baseline characteristics are presented in Table 1.

**TABLE 1:** Comparison of baseline characteristics between the stable group and the rapid-growth group.

Characteristic	Stable group (N = 51)	Growth group (N = 30)	p-value
Age, years	68.00 [63.50, 71.50]	66.00 [63.00, 72.00]	0.761
Male	47 (92.16)	26 (86.67)	0.679
Smoking history	11 (21.57)	15 (50.00)	0.016
Drinking history	7 (13.73)	8 (26.67)	0.249
Hypertension	19 (37.25)	8 (26.67)	0.464
Diabetes	2 (3.92)	2 (6.67)	0.984
CHD	8 (15.69)	9 (30.00)	0.213
Prior IS	4 (7.84)	4 (13.33)	0.679
Prior surgery	16 (31.37)	8 (26.67)	0.845
SBP, mmHg	134.00 [124.00, 141.00]	137.00 [127.25, 143.75]	0.417
DBP, mmHg	78.00 [70.00, 85.50]	80.00 [69.50, 87.50]	0.534
Fibrinogen, g/L	3.72 [2.97, 4.35]	3.77 [3.04, 4.48]	0.899
FBG, mg/dL	99.72 [90.36, 111.51]	105.17 [94.16, 113.59]	0.291
Creatinine, mmol/L	74.90 [64.51, 85.11]	76.49 [66.05, 87.34]	0.671
C-reactive protein	3.10 [1.90, 4.38]	3.30 [1.77, 4.71]	0.833
LDL-C, mmol/L	91.22 [71.82, 113.05]	92.57 [69.59, 105.68]	0.487
D-Dimer, mg/dL	167.59 [107.28, 210.75]	150.18 [116.75, 198.53]	0.973
Aneurysm length, mm	70.60 [56.35, 87.00]	82.40 [70.17, 88.20]	0.07
Neck length, mm	39.10 [23.30, 53.25]	30.90 [25.50, 41.92]	0.246
Maximum diameter of AAA, mm	41.70 [35.45, 45.25]	43.70 [38.05, 48.48]	0.159

Categorical variables are reported as frequency and percentage (n,%).

Continuous variables are reported as median and interquartile range (med [Q1 − Q3]).

CHD, coronary atherosclerotic heart disease; IS, ischemic stroke; SBP, systolic blood pressure; DBP, diastolic blood pressure; FBG, fasting blood glucose; LDL-C, low-density lipoprotein cholesterol; AAA, abdominal aortic aneurysm.

### CTA images containing abdominal aorta classification

**FIGURE 4:**
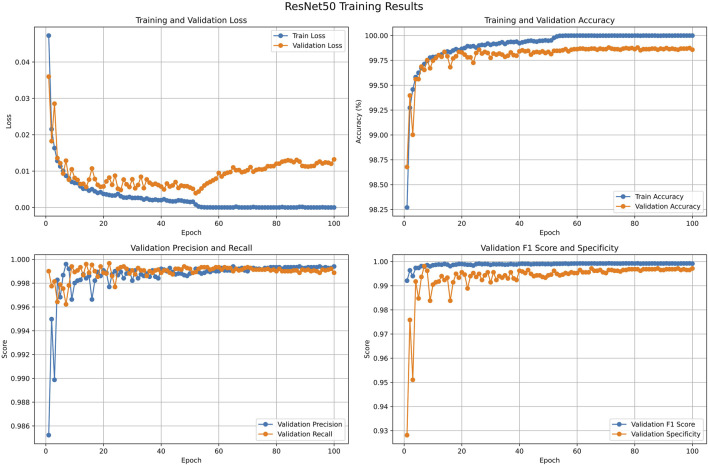
Results of the ResNet50 model for classifying CTA images containing the abdominal aorta. CTA, computed tomography angiography.

The CTA data were randomly split into training, validation, and test sets in a ratio of 8:1:1 at the patient level, which is displayed in the Supplementary Table S1. We compared eight models suitable for image classification, mainly to identify CTA images containing the abdominal aorta. The results of the model comparison are shown in Table 2. Among these models, the ResNet50 model performed best, with an accuracy (ACC) and F1-score of 99.86% and 99.91%, respectively. The ResNet50 model classified the CTA images suitable for the next step, that is, abdominal aorta detection, and the results of model training and validation are shown in Figure 4.

**TABLE 2:** Performance analysis of different models for classifying CTA images containing the abdominal aorta on the test set.

Model	Accuracy (%)	F1-score (%)	Precision (%)	Recall (%)	AUC (%)
VGG16	82.74	90.55	82.74	100.00	50.00
ResNet18	99.85	99.91	99.93	99.89	99.94
ResNet50	99.86	99.91	99.93	99.89	99.97
ResNet101	99.82	99.89	99.90	99.88	99.92
DenseNet121	99.84	99.90	99.90	99.91	99.95
ViT-B/16	94.97	96.97	96.79	97.14	98.19
MedViT	85.52	91.65	87.66	96.02	82.44
MedMamba	99.82	99.89	99.88	99.91	100.00

CTA, computed tomography angiography; AUC, area under the curve.

### Detecting and classifying the abdominal aorta

**FIGURE 5:**
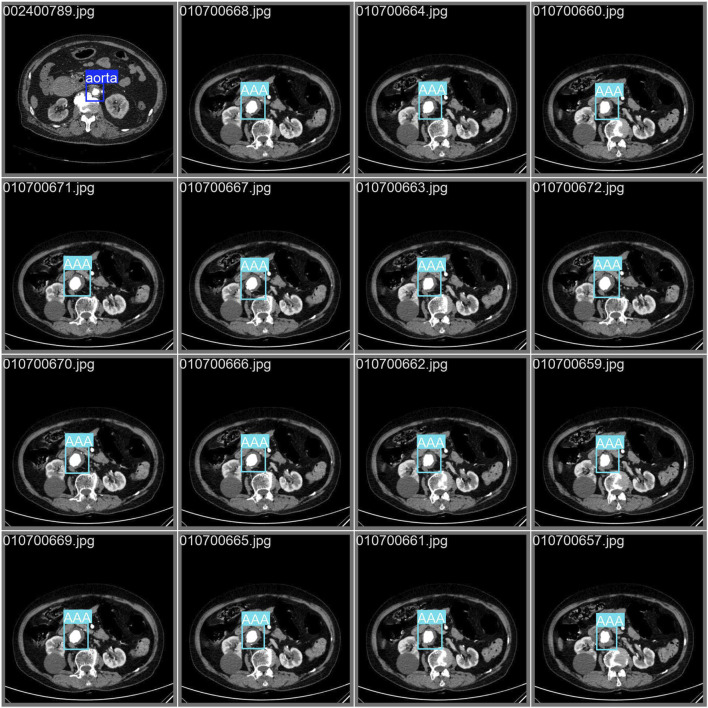
Validation for classifying the normal aorta and abdominal aortic aneurysm of the YOLOv11 model on CTA images. YOLO, You Only Look Once; CTA, computed tomography angiography.

**FIGURE 6:**
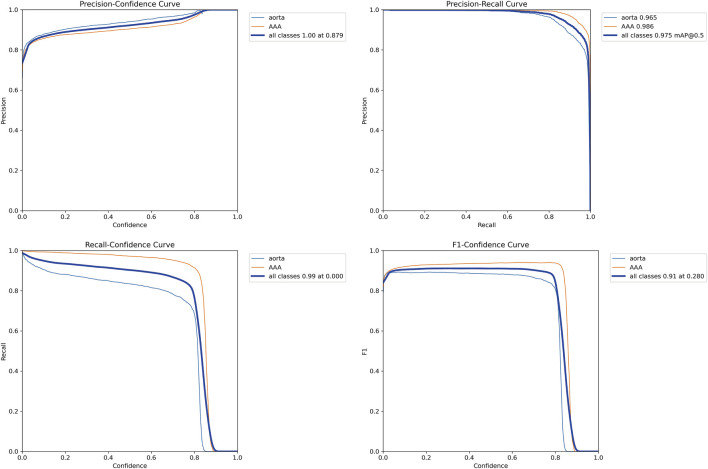
Performance curves of the YOLOv11 model for detecting and classifying the abdominal aorta. YOLO, You Only Look Once.

**FIGURE 7:**
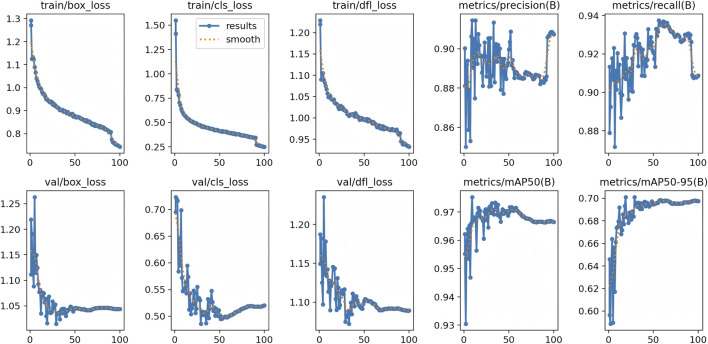
Training and validation losses with performance metrics of the YOLOv11 model for detecting and classifying the abdominal aorta. YOLO, You Only Look Once.

A total of 31,457 CTA images containing the abdominal aorta were trained with 100 epochs and were completed in 13.479 h. The YOLOv11 model detected the abdominal aorta and classified the normal aortae and AAAs with the highest precision (p = 0.902), which indicates that the model has a strong ability to correctly identify true positives. Its robust performance across four YOLO models is demonstrated in Table 3. This implementation demonstrates a high effectiveness in distinguishing between the normal aorta and the AAA, and the validation set is shown in Figure 5. The model correctly identifies the regions of interest and highlights them with bounding boxes in distinct colors. Figure 6 depicts key performance metrics of the classification result between the normal aorta and AAA. The precision–confidence curve (top left) shows how precision varies with confidence thresholds, indicating consistently high precision near the threshold of 0.902 for all categories. The precision–recall curve (top right) illustrates the relationship between precision and recall, demonstrating stable performance, with the overall mAP50 reaching 0.975. The recall–confidence curve (bottom left) highlights a gradual decline in recall as confidence thresholds increase, reflecting the trade-off between higher confidence and recall. Lastly, the F1–confidence curve (bottom right) provides insights into the harmonic means of precision and recall, maintaining a high F1-score across confidence levels. These curves collectively validate the model’s robustness and balanced performance across the different classification groups. Figure 7 illustrates how the model performed during training and validation over 100 epochs. In the top row, we see the training losses for bounding box regression, classification, and distribution focal loss. All these losses show a steady decline, which suggests that the model is being effectively optimized. Additionally, metrics like precision, recall, mAP50, and mAP50-95 consistently improve, reaching high levels by the end of training. The results of the other three YOLO models are presented in the supplementary materials.

**TABLE 3:** Performance comparison of four YOLO models.

Model	Training time (h)	mAP50	mAP50-95	Precision	Recall
YOLOv5	13.626	0.970	0.697	0.895	0.928
YOLOv8	13.515	0.971	0.690	0.881	0.926
YOLOv10	14.521	0.972	0.692	0.889	0.926
YOLOv11	13.479	0.975	0.704	0.902	0.924

YOLO, You Only Look Once; mAP, mean average precision.

### Feature fusion model

**FIGURE 8:**
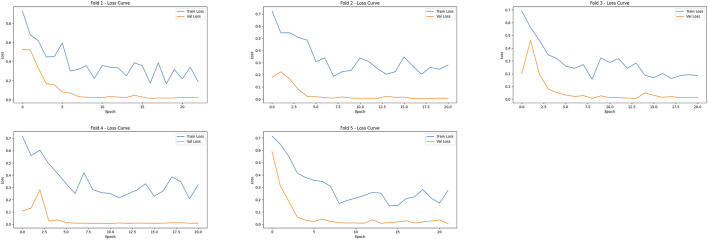
Training and validation losses of the feature fusion model based on the MedMamba backbone.

**FIGURE 9:**
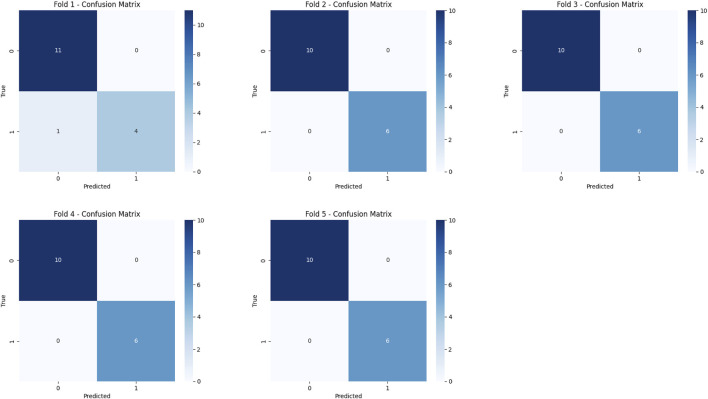
Confusion matrices for the feature fusion model based on the MedMamba backbone.

**FIGURE 10:**
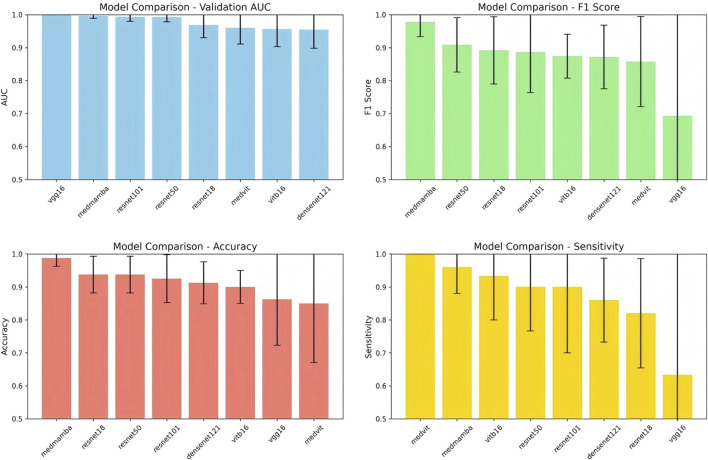
Performance comparisons of the MedMamba backbone with the traditional models to predict abdominal aortic aneurysm growth.

A total of 15,704 CTA images of the local abdominal aorta were provided by the YOLOv11 model, which were utilized for the feature fusion model. Among the eight models, the MedMamba was considered the most suitable backbone for further feature fusion to predict AAA growth, which achieved an accuracy of 98.75% and an F1-score of 97.78%. The training and validation loss curves in Figure 8 indicate that the model has converged. The confusion matrices suggest that misclassifications are rare, reinforcing the model’s reliability, as shown in Figure 9. Compared with the VGG16, ResNet18, ResNet50, ResNet101, DenseNet121, ViT-B/16, and MedViT models, the feature fusion model based on MedMamba achieved the best performance, detailed in Table 4 and Figure 10. The training and validation loss curves and confusion matrices of feature fusion models based on seven other backbones are shown in the supplementary materials.

**TABLE 4:** Performance analysis of the MedMamba model and the traditional models to predict abdominal aortic aneurysm growth.

Backbone	Accuracy (%)	F1-score (%)	Sensitivity (%)	Specificity (%)	AUC (%)
VGG16	86.25	69.33	63.33	100.00	100.00
ResNet18	93.75	89.18	82.00	100.00	96.88
ResNet50	93.75	90.85	90.00	96.36	99.27
ResNet101	92.50	88.66	90.00	94.18	99.33
DenseNet121	91.25	87.19	86.00	94.36	95.45
ViT-B/16	90.00	87.43	93.33	88.00	95.67
MedViT	85.00	85.79	100.00	76.18	96.00
MedMamba	98.75	97.78	96.00	100.00	99.64

AUC, area under the curve.

### Experiments to explore attention modules on model performance

We compared the prediction performance effects of applying different attention modules based on the MedMamba model. From the analysis of Table 5, the MHA mechanism has excellent performance compared to the other attention modules. When applying the MHA mechanism for feature fusion, we compared the effects of 4 heads, 8 heads, 16 heads, and 32 heads on the model results, as shown in Table 6. The 8-head self-attention mechanism achieved the optimal balance and resulted in the best prediction of AAA growth.

**TABLE 5:** Evaluation of different attention modules on CTA images to predict abdominal aortic aneurysm growth.

Attention	AUC (%)	Accuracy (%)	F1-score (%)	Sensitivity (%)	Specificity (%)
MedMamba + MHA	99.64	98.75	97.78	96.00	100.00
MedMamba + CBAM	100.00	93.75	91.32	90.00	96.00
MedMamba + Channel	99.33	91.25	87.49	86.67	94.36
MedMamba + Cross	98.64	86.25	72.51	65.33	98.00
MedMamba + SE	100.00	87.50	74.18	64.00	100.00
MedMamba + GAM	96.52	88.75	78.94	68.00	100.00

CTA, computed tomography angiography; MHA, multi-head self-attention; CBAM, convolutional block attention module; SE, Squeeze-and-Excitation module; GAM, global attention mechanism.

**TABLE 6:** Evaluating the number of heads of multi-head self-attention for predicting abdominal aortic aneurysm growth.

Number of heads	AUC (%)	Accuracy (%)	F1-score (%)	Sensitivity (%)	Specificity (%)
4	100.00	96.25	93.18	88.67	100.00
8	99.64	98.75	97.78	96.00	100.00
16	100.00	92.50	91.13	100.00	88.36
32	100.00	92.50	90.29	93.33	92.00

AUC, area under the curve.

### Model interpretability and visualization

**FIGURE 11:**
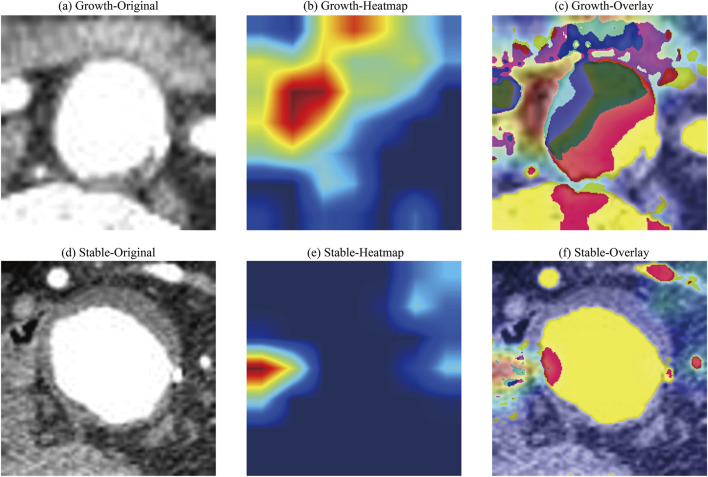
Representative Grad-CAM visualizations for a rapid-growth AAA case **(a–c)** and a stable AAA case **(d–f)**. **(a–d)** Original CTA image slices. **(b–e)** Gradient-weighted class activation mapping (Grad-CAM) heatmaps. **(c–f)** Heatmaps superimposed on the original images. The model’s attention in the rapid-growth case is localized to the perivascular region, while such focused activation is absent in the stable case. Grad-CAM, gradient-weighted class activation mapping; AAA, abdominal aortic aneurysm; CTA, computed tomography angiography.

Grad-CAM was used to generate heatmaps that visually interpreted the model’s reasoning process by highlighting regions within the input CTA images that most strongly influenced its classification decision for ruptured abdominal aortic aneurysm (RAAA), as shown in Figure 11. These visual results indicate that the model consistently focuses on anatomically significant areas, such as suggesting focal enhancement and activation in the perivascular regions, which have a strong correlation with the established imaging markers of AAA growth. Unexpectedly, the areas of thickened blood vessel walls that might reflect vascular inflammatory responses and characteristics of atherosclerosis were not activated.

## Discussion

The accurate prediction of small AAA growth remains a significant challenge in clinical practice. Current management, guided by consensus guidelines, primarily relies on periodic surveillance using the maximum aortic diameter as a surrogate for rupture risk. While practical, this conventional approach often fails to capture the complex, multifactorial nature of aneurysm progression, which involves a dynamic interplay of biomechanical stress, chronic inflammation, and proteolytic degradation. This study establishes a novel, automated deep learning framework that integrates ResNet50, YOLOv11, and MedMamba to predict the rapid expansion of asymptomatic small AAAs directly from CTA images and clinical data. The end-to-end pipeline achieved an exceptionally high predictive performance, with an accuracy of 98.75% and an AUC of 99.64% in our cohort of 81 asymptomatic patients. Critically, despite the cohort size, this performance was consistent across rigorous internal validation, demonstrating the substantial potential of leveraging these architectures to automate the entire workflow, from image screening and precise, bounding-box-level aneurysm localization to multimodal feature fusion. This design crucially circumvents the manual segmentation and expert-dependent feature engineering required by traditional radiomics and hemodynamic models (Ristl et al., 2021; Kontopodis et al., 2022; Kim et al., 2023; Wang et al., 2023; Starck et al., 2025). This performance likely stems from both the architectural synergy between detection and sequence modeling components and the framework’s capacity to learn discriminative features directly from raw data, potentially capturing subtle prognostic image patterns that may serve as surrogates for underlying pathophysiological processes, such as inflammation or wall stress, which are lost in manual preprocessing. This study thereby suggests a new performance benchmark for AAA progression prediction and highlights a clinically translatable, scalable pathway for future decision-support tools.

The strong predictive performance of our framework is likely attributable to the synergistic design of its constituent architectures, each addressing specific limitations in conventional AAA analysis. This integrated design effectively establishes a fully automated pipeline that progresses from the identification of aortic images (ResNet50) to precise, bounding-box-level localization of the aneurysm (YOLOv11), and culminates in the fusion of imaging features based on the MedMamba backbone with clinical data. A key advantage of this design is its circumvention of manual segmentation, a step typically required in radiomics and biomechanical modeling, which can introduce observer variability and hinder scalability. The choice of YOLOv11 for localization ensures that subsequent feature extraction is focused exclusively on the pathologically relevant region, minimizing contamination from adjacent anatomic structures. However, the most substantial performance improvements, as evidenced by our comparative ablation studies, appear to originate from the feature fusion stage. Here, the MedMamba backbone was selected for its potential to model long-range dependencies across sequential CTA slices, potentially capturing a more global spatiotemporal context of the aneurysms than standard CNNs. This was complemented by the optimized 8-head MHA mechanism, which acts as a sophisticated information integrator. Our ablation studies (Table 6) demonstrate that this specific configuration offered an optimal balance, enabling the model to integrate multi-scale features, from local textural variations to broader morphological changes. The synergy between MedMamba’s sequential processing and MHA’s cross-modal alignment may allow the model to learn a hierarchical representation that captures morphological and textural heterogeneities, which, although recognized in the literature as markers of progression, are elusive to manual quantification. Consequently, by moving beyond the simplistic reliance on maximum diameter, which is the current cornerstone of clinical guidelines (Isselbacher et al., 2022), our framework suggests a path toward a more holistic, pathophysiologically grounded assessment of AAA progression risk. This could potentially enable more personalized surveillance strategies, ensuring that high-risk patients receive timely intervention while reducing unnecessary imaging for those with stable disease.

When contextualizing our results within the existing landscape of AAA prediction, our automated deep learning framework addresses several persistent methodological challenges. The field has progressively identified key prognostic indicators underpinned by specific pathophysiological mechanisms: for instance, the intraluminal thrombus is not merely a structural feature but an active source of proteolytic activity and wall inflammation that fuels progression (Zhu et al., 2020); radiomic signatures of the perivascular adipose tissue (Lv et al., 2024) may reflect a paracrine, inflammatory “outside-to-inside” signaling pathway; and the role of vascular calcification (Klopf et al., 2022) involves complex biomechanical alterations to wall stress distribution. However, translating these mechanistic insights into clinical tools has been constrained by methodology. Extracting these features typically relies on expert-dependent manual segmentation, a process that not only introduces variability but may also restrict analysis to a predefined set of human-engineered features, as seen in radiomics studies (Kontopodis et al., 2022; Wang et al., 2023), potentially missing subtler, synergistic patterns. Further compounding this, other approaches seek to integrate more complex data, such as combining anatomical imaging with hemodynamic simulations derived from reconstructed geometries to estimate wall stress (Kim et al., 2023). While physiologically insightful, the reliance on manual segmentation and complex multi-physics modeling poses a significant barrier to clinical scalability. In contrast, our ResNet50-YOLOv11-MedMamba framework addresses these bottlenecks by learning directly from raw data. The pipeline automatically transitions from CTA images to a prediction. We hypothesize that by doing so, the model does not merely “see” thrombus or calcification in a conventional sense but may learn complex imaging signatures that are surrogate markers for the underlying inflammatory and biomechanical processes these established features represent. This end-to-end strategy is posited to allow the model to learn a rich, hierarchical representation that can capture the prognostic information contained in the complex interplay of these mechanisms, without being constrained to predefined feature sets. This capability is a plausible explanation for its competitive performance when compared to models utilizing manually segmented features (Kontopodis et al., 2022; Wang et al., 2023) or those relying on a more limited set of traditional parameters (Ristl et al., 2021). Consequently, our work suggests a scalable pathway that leverages deep learning to integrate the pathophysiological complexity of AAA progression into a unified predictive assessment.

The biological plausibility of our model’s predictions was further interrogated using SHAP analysis and Grad-CAM visualizations. SHAP analysis confirmed that the model’s decision-making aligns with established clinical knowledge (Ullery et al., 2018), identifying smoking history, larger baseline aneurysm diameter, and advanced age as the most influential features, which are all well-documented risk factors for AAA progression. This concordance lends critical face validity, demonstrating that the model rationally leverages clinically pertinent information. The interpretation of Grad-CAM visualizations, however, must be contextualized within our model’s architecture. Given that the YOLOv11 pipeline first precisely localizes the aneurysm, Grad-CAM does not function in a primary diagnostic localization role. Instead, it elucidates which sub-regions within and surrounding the delineated aneurysm most strongly drive the prediction. A striking and consistent pattern emerged: in cases of rapid expansion, the model’s attention was frequently mapped not to the aortic wall itself, but to the adjacent perivascular adipose tissue (PVAT). We hypothesize that this pattern may indicate the model’s sensitivity to imaging signatures of perivascular inflammation. PVAT is a recognized secretory organ, modulating vascular inflammation, which is a cornerstone of AAA pathogenesis, via the paracrine release of adipokines and cytokines. It is plausible that our model detects subtle textural or radiodensity alterations within this tissue, capturing a novel imaging biomarker of a pro-inflammatory state. This hypothesis conceptually bridges the imaging findings with the systemic risk profiles highlighted by SHAP (e.g., smoking). We emphasize that this remains a hypothesis-generating observation that warrants future validation through direct correlation with histopathological analysis or specific inflammatory serological markers. Collectively, these explainability techniques present a coherent, biphasic narrative. The model initially anchors its decision in fundamental, patient-specific clinical risks (SHAP) and subsequently appears to refine its prognostic assessment by integrating a localized, imaging-based evaluation of the perivascular milieu (Grad-CAM). This synergy suggests that our framework transcends a mere “black-box” predictor, instead synthesizing established clinical wisdom with potential novel imaging biomarkers to enable a more holistic and mechanistically informed risk stratification for AAA progression.

A recent study by Oh et al. (2025) also developed a deep learning model for predicting AAA progression, providing a valuable benchmark for the field. Our work complements and extends this effort in several key aspects that may contribute to the differing performance profiles. First, the clinical targets differ. Our model was trained to predict rapid growth defined as ≥0.5 cm per 6 months, a threshold directly aligned with guideline recommendations for considering intervention. In contrast, Oh et al. employed a threshold of 2.5 mm/year. Predicting this more abrupt, clinically decisive growth event represents a distinct and potentially more challenging task, which may partly account for the differing performance metrics observed. Second, the feature-extraction paradigms diverge. Their approach relied on manually segmenting aortic geometries to derive predefined radiomic features, a methodologically rigorous but inherently limited process. Our fully automated YOLOv11-based localization, while providing less geometrically precise segmentation, preserves and analyzes the entire periaortic imaging context. This approach may have enabled our model to capture the prognostic information from the perivascular environment (Lv et al., 2024), as directly suggested by our Grad-CAM findings, which is typically excluded during manual contouring. Therefore, our framework explores a different trade-off, prioritizing automated, context-aware feature discovery over manual, lesion-specific measurement. When viewed together, these studies illustrate two parallel and complementary paths forward: one focused on creating robust, manually verified models from large cohorts, and the other pushing the boundaries of fully automated performance.

Beyond the methodological comparisons within the domain of CT-based modeling, our study also lays the groundwork for future integration with emerging, non-invasive biomarkers that probe different aspects of AAA pathophysiology. For instance, circulating biomarkers such as soluble glycoprotein VI (Benson et al., 2024) and specific microRNAs (Thanigaimani et al., 2022) have shown promise in reflecting platelet activity and cellular stress associated with aneurysm growth. Similarly, advanced MRI techniques, including MR elastography to assess wall stiffness (Dong et al., 2022) and 4D flow to quantify wall shear stress (Trenti et al., 2022), provide unique biomechanical and functional insights not directly accessible by standard CTA. While these modalities are not yet part of routine clinical practice for AAA surveillance, they represent a rich source of complementary data. The automated architecture presented in this study is, in principle, amenable to incorporating such multimodal inputs in the future. A compelling long-term goal would be to fuse our automated imaging-derived predictions with these serological and functional biomarkers, potentially creating a supremely robust, multi-parametric risk assessment tool that captures the biological, morphological, and biomechanical drivers of AAA progression in concert.

## Limitations

Our study has several limitations that should be considered. First, the performance of our fully automated framework was evaluated on a single-center cohort with a limited sample size. Although we employed rigorous internal validation strategies, including 5-fold cross-validation and comprehensive data augmentation, to ensure robustness and mitigate overfitting, the generalizability of the model requires confirmation in larger, multi-center populations. Our findings may thus be viewed as complementary to recent large-scale, multi-center efforts, such as the study by Oh et al. (2025), which established a robust benchmark for generalizability. While their study exemplifies a pathway toward clinical deployment, our work explores the potential performance gains achievable with a novel architecture and full automation, suggesting a promising direction for future technical development. Second, our cohort was predominantly male, which aligns with the epidemiological prevalence of AAA but limits the model’s applicability to female patients, a population with recognized differences in rupture risk. Third, the retrospective design precluded the inclusion of genetic or novel biomarker data. Finally, while our explainability analyses provide strong, hypothesis-generating insights into the model’s decision-making, for instance, by highlighting the role of perivascular tissue, a comprehensive mapping of the full hierarchy of deep features to their specific biological correlates remains a key goal for future research. This includes linking these features to underlying biological processes such as specific inflammatory cell infiltrates or proteolytic activity levels. These considerations collectively outline a clear and logical path for future research. The initial performance achieved here justifies the next critical step: a prospective, multi-center validation study. Such an endeavor would be essential to confirm generalizability and would also provide a platform to integrate imaging-based predictions with multimodal data. Building upon the automated architecture presented here, future work that leverages large, diverse cohorts will be crucial to translating these technical advances into clinically reliable tools for personalized AAA management.

## Conclusion

This study developed and validated a novel, end-to-end deep learning framework, ResNet50–YOLOv11–MedMamba, for predicting the growth of small asymptomatic AAAs. By integrating clinical data with automatically extracted imaging features from CTA, our model achieved a high predictive performance (98.75% accuracy, 97.78% F1-score) in internal validation, outperforming several classical benchmarks. The framework’s design, which leverages YOLOv11 for precise lesion localization and an 8-head MHA mechanism for effective feature fusion, demonstrates the feasibility of fully automating growth prediction. Future work, focused on multi-center prospective validation and the inclusion of biomechanical markers, is warranted to confirm these findings and pave the way for more personalized, image-based surveillance strategies.

## Data Availability

The raw data supporting the conclusions of this article will be made available by the authors, without undue reservation.
