# Supplementary material for: Predicting the growth of asymptomatic small abdominal aortic aneurysms (AAA) based on deep learning
Source: Front Physiol. 2026 Jan 27;16:1704428. doi: 10.3389/fphys.2025.1704428 (PMC12886037; doi:10.3389/fphys.2025.1704428)

Supplements.

Figure 1. Results of the VGG16 model for classifying CTA images containing the abdominal aorta. CTA: Computed tomography angiography.


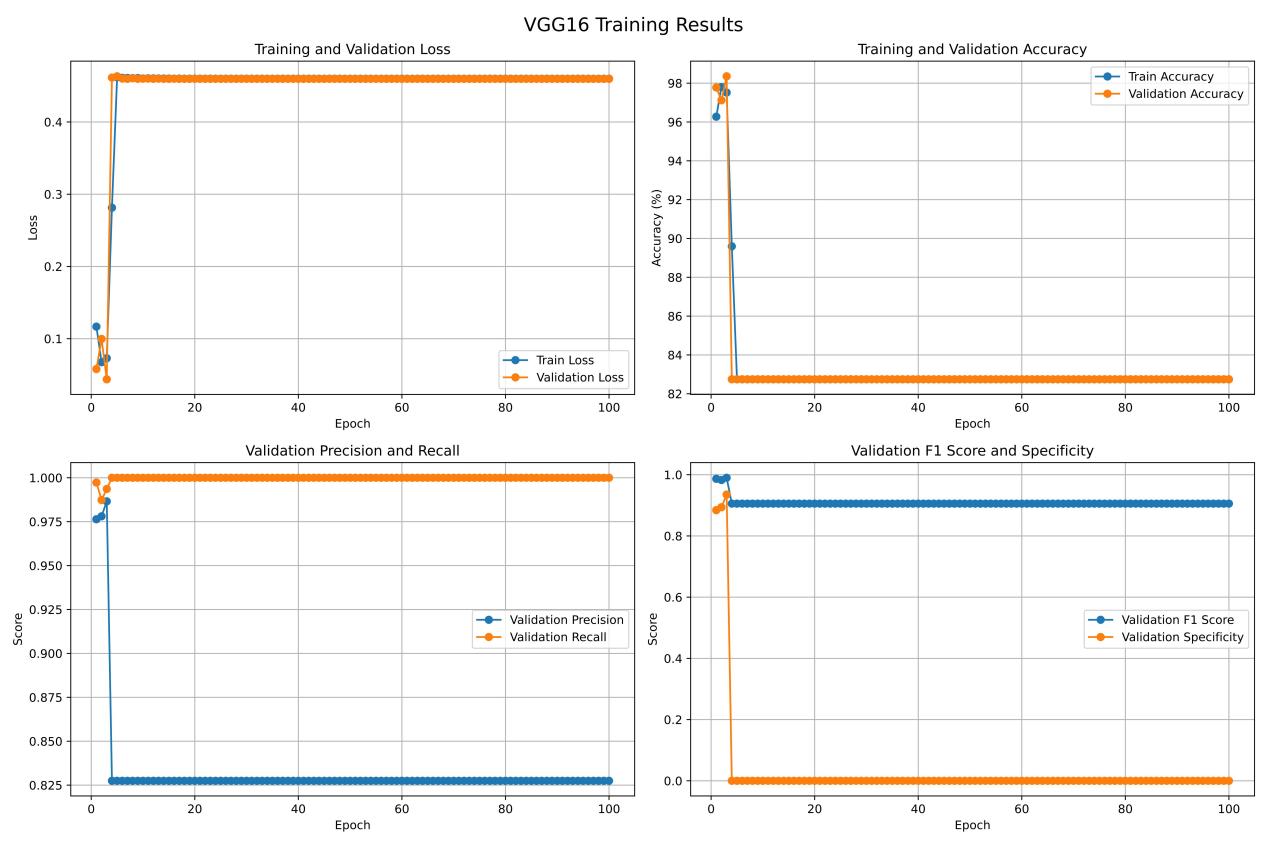


Figure 2. Results of the ResNet18 model for classifying CTA images containing the abdominal aorta. CTA: Computed tomography angiography.


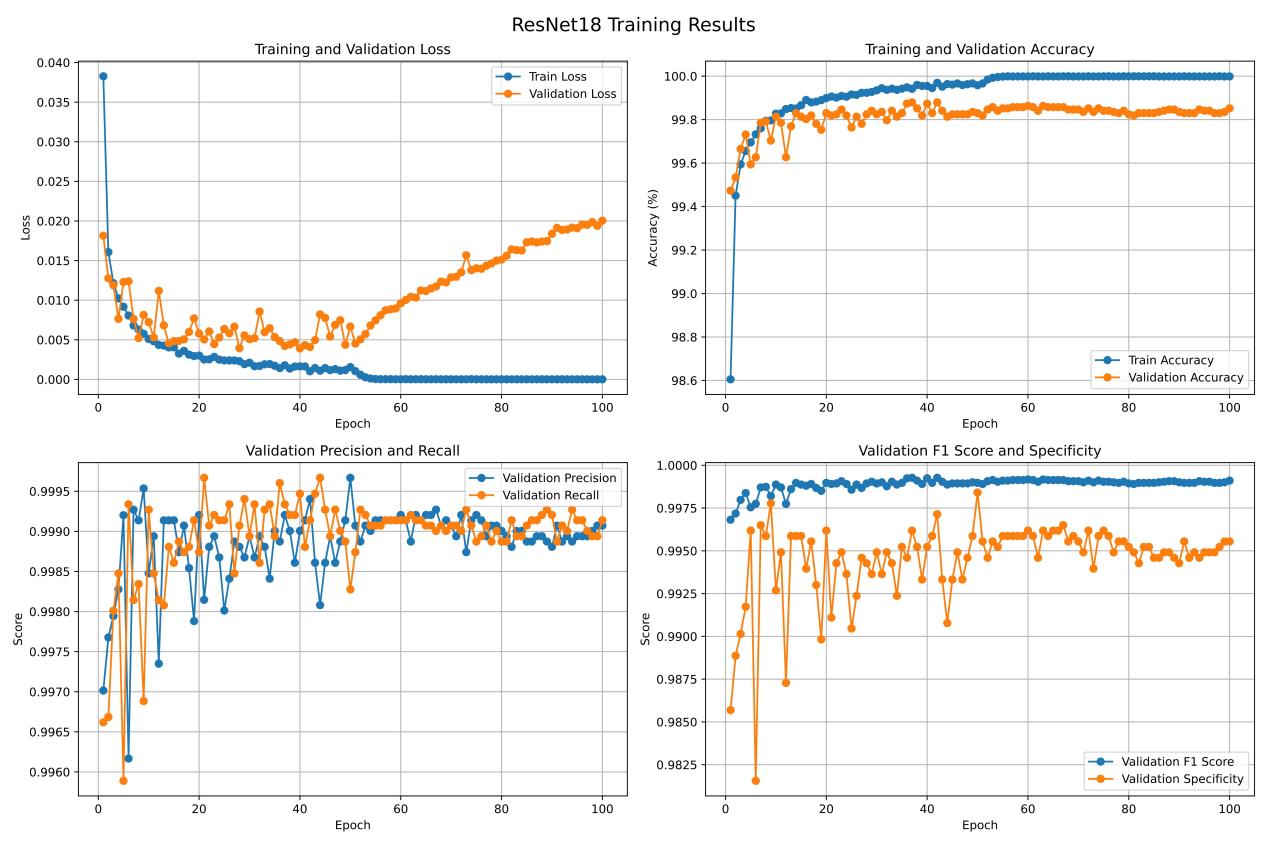


Figure 3. Results of the ResNet101 model for classifying CTA images containing the abdominal aorta. CTA: Computed tomography angiography.


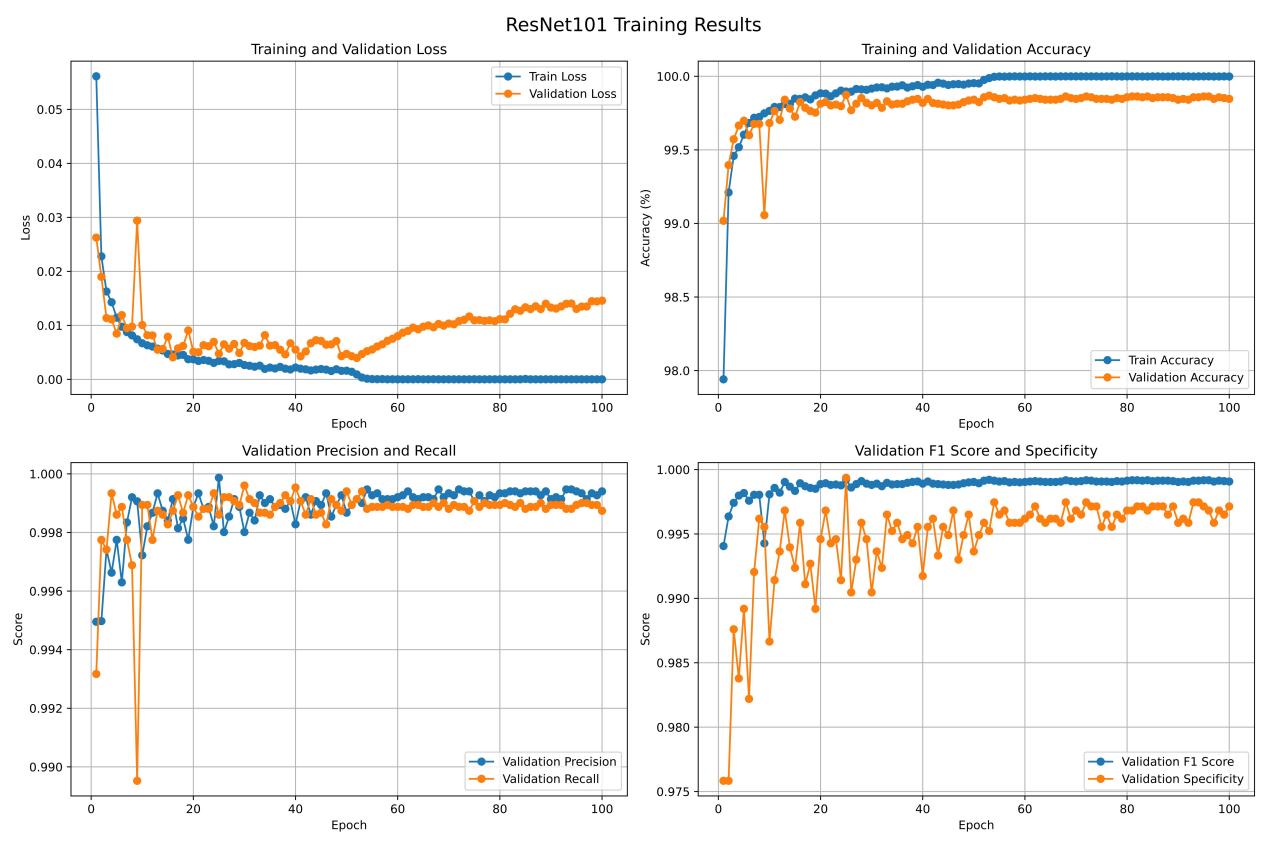


Figure 4. Results of the DenseNet121 model for classifying CTA images containing the abdominal aorta. CTA: Computed tomography angiography.


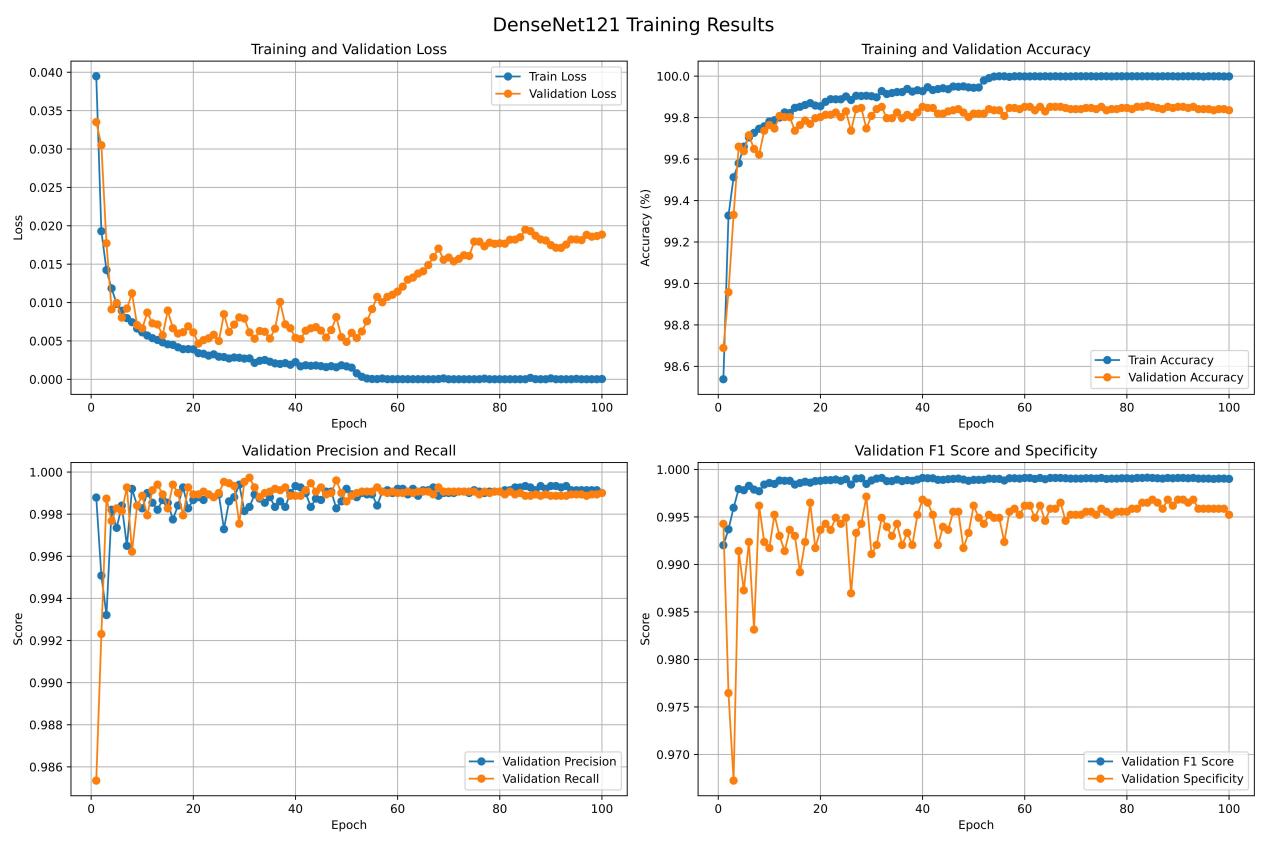


Figure 5. Results of the ViT-B/16 model for classifying CTA images containing the abdominal aorta. CTA: Computed tomography angiography.


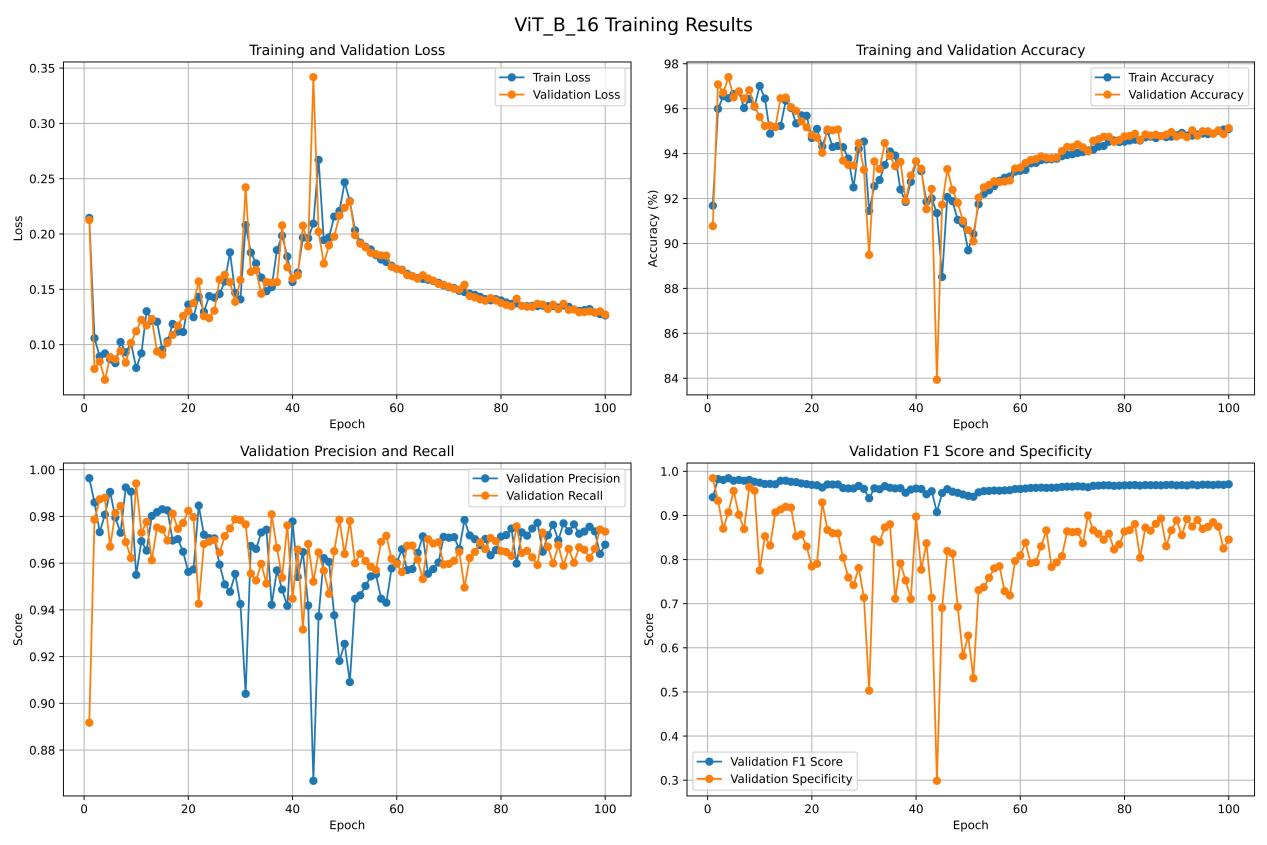


Figure 6. Results of the MedViT model for classifying CTA images containing the abdominal aorta. CTA: Computed tomography angiography.


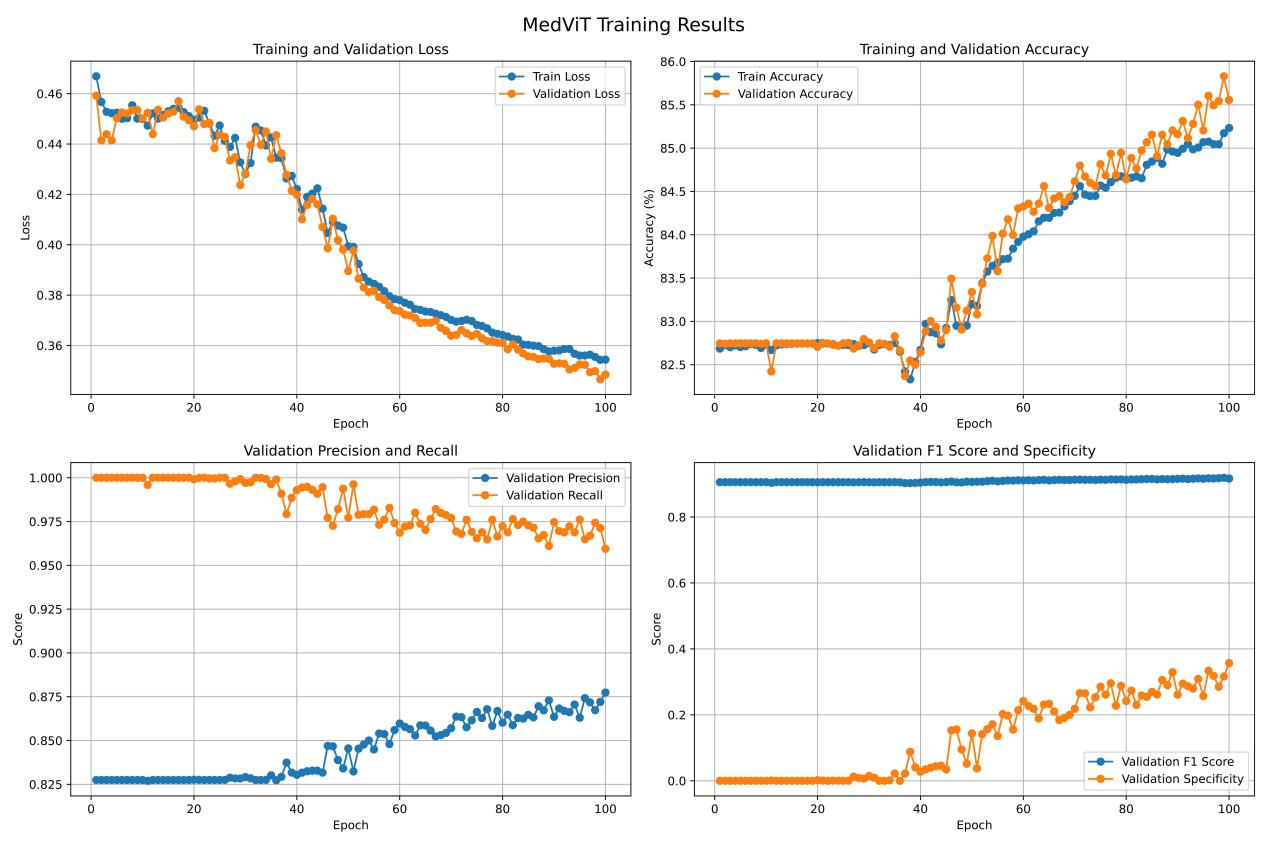


Figure 7. Results of the MedMamba model for classifying CTA images containing the abdominal aorta. CTA: Computed tomography angiography.


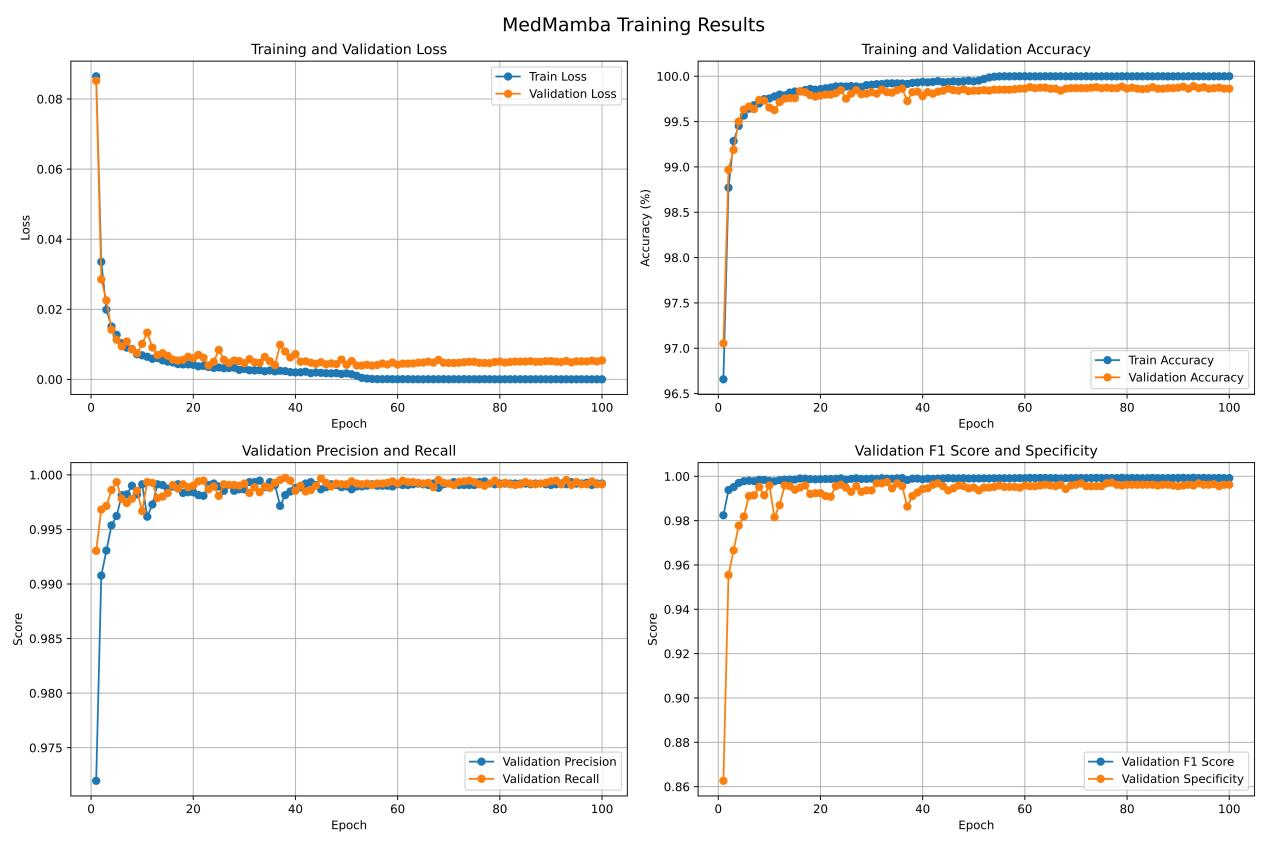


Figure 8. Performance comparisons of the ResNet50 with the traditional models to classify CTA images containing the abdominal aorta.


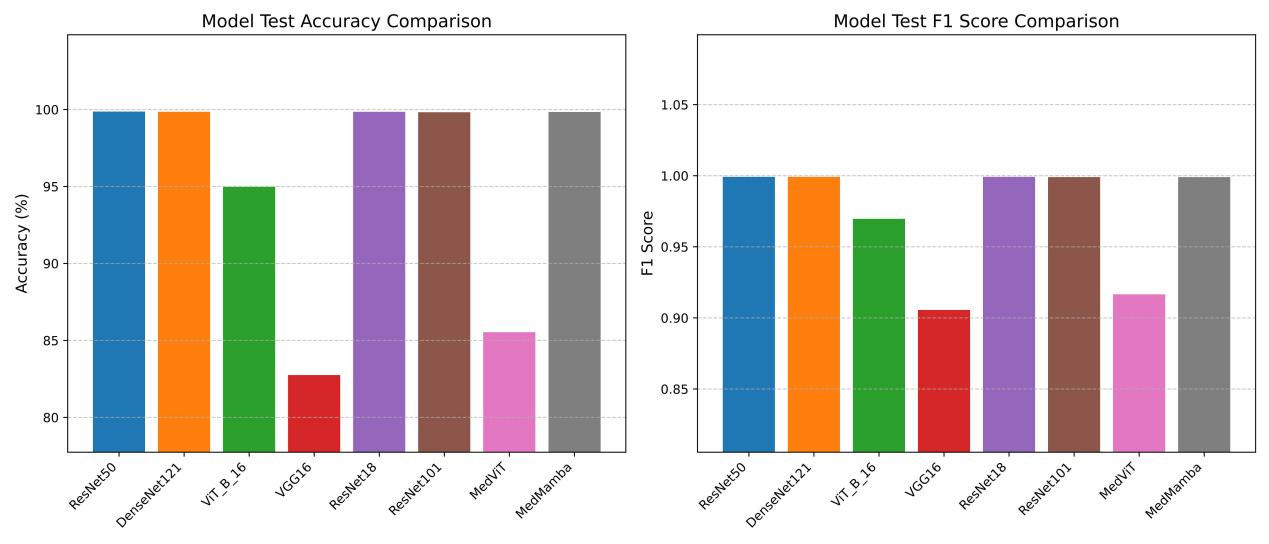


Figure 9. Training for classifying the normal aorta and abdominal aortic aneurysm of the YOLOv5 model on CTA images. YOLO: You Only Look Once; CTA: Computed tomography angiography.


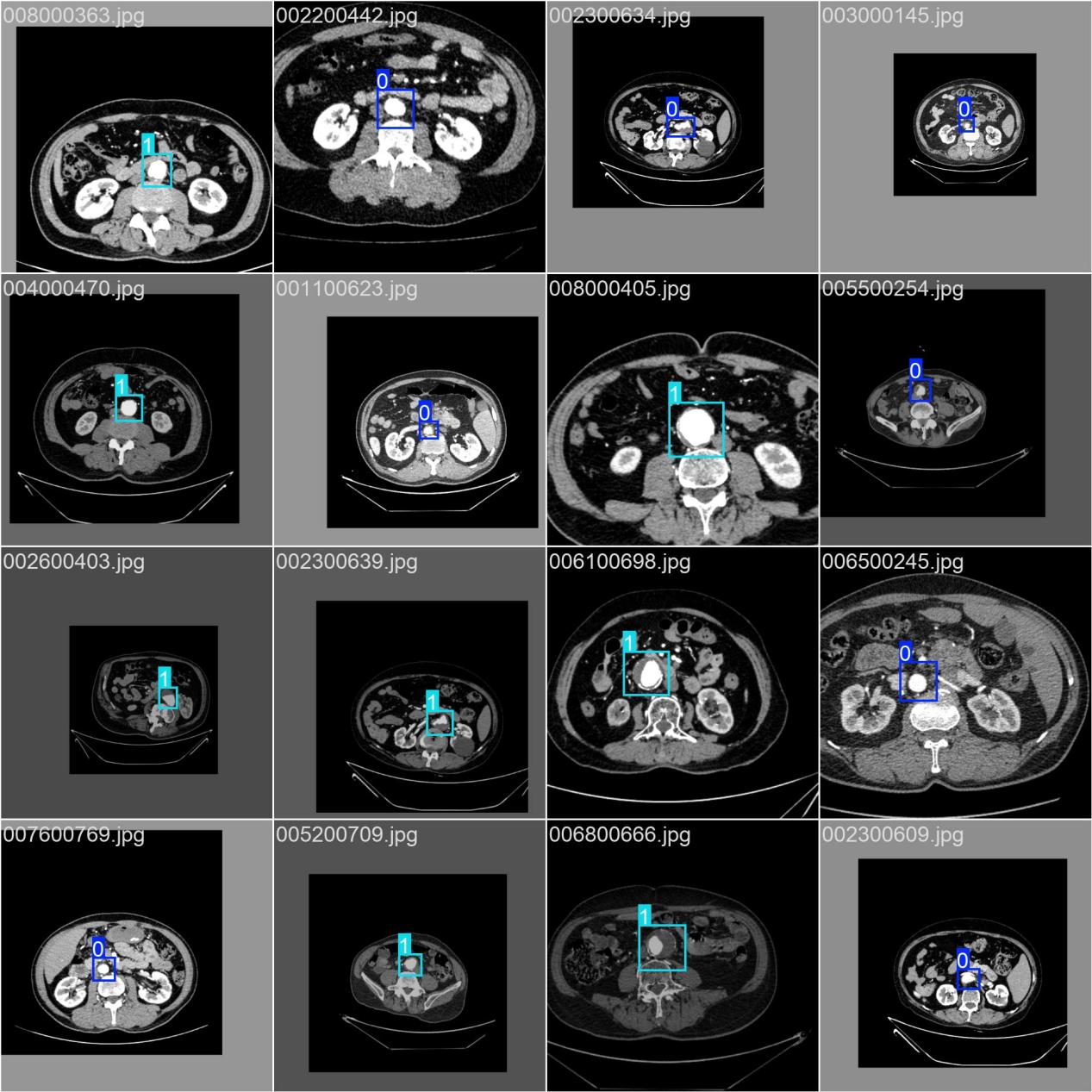


Figure10. Validation for classifying the normal aorta and abdominal aortic aneurysm of the YOLOv5 model on CTA images. YOLO: You Only Look Once; CTA: Computed tomography angiography.


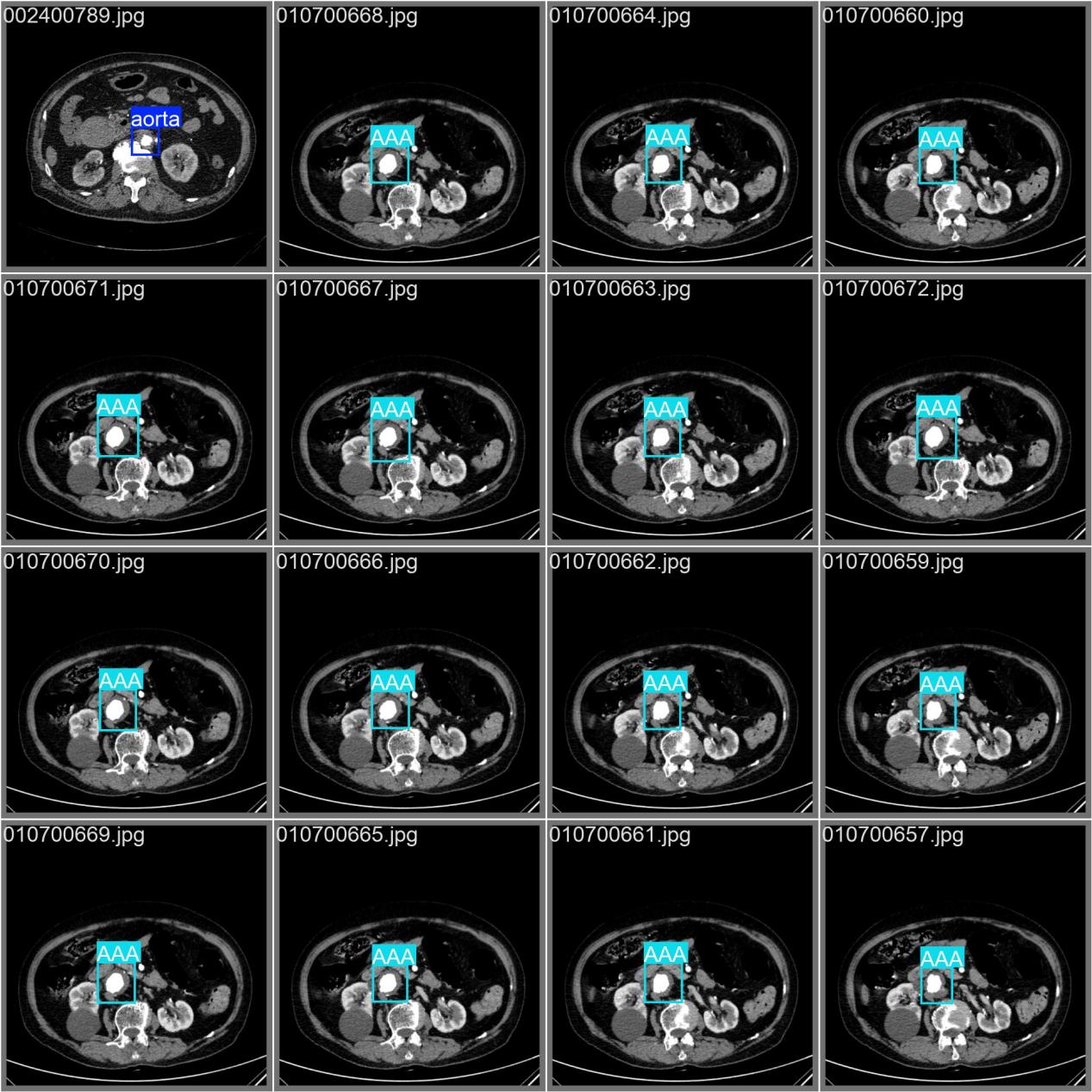


Figure 11. Performance curves of the YOLOv5 model for detecting and classifying the abdominal aorta. YOLO: You Only Look Once.


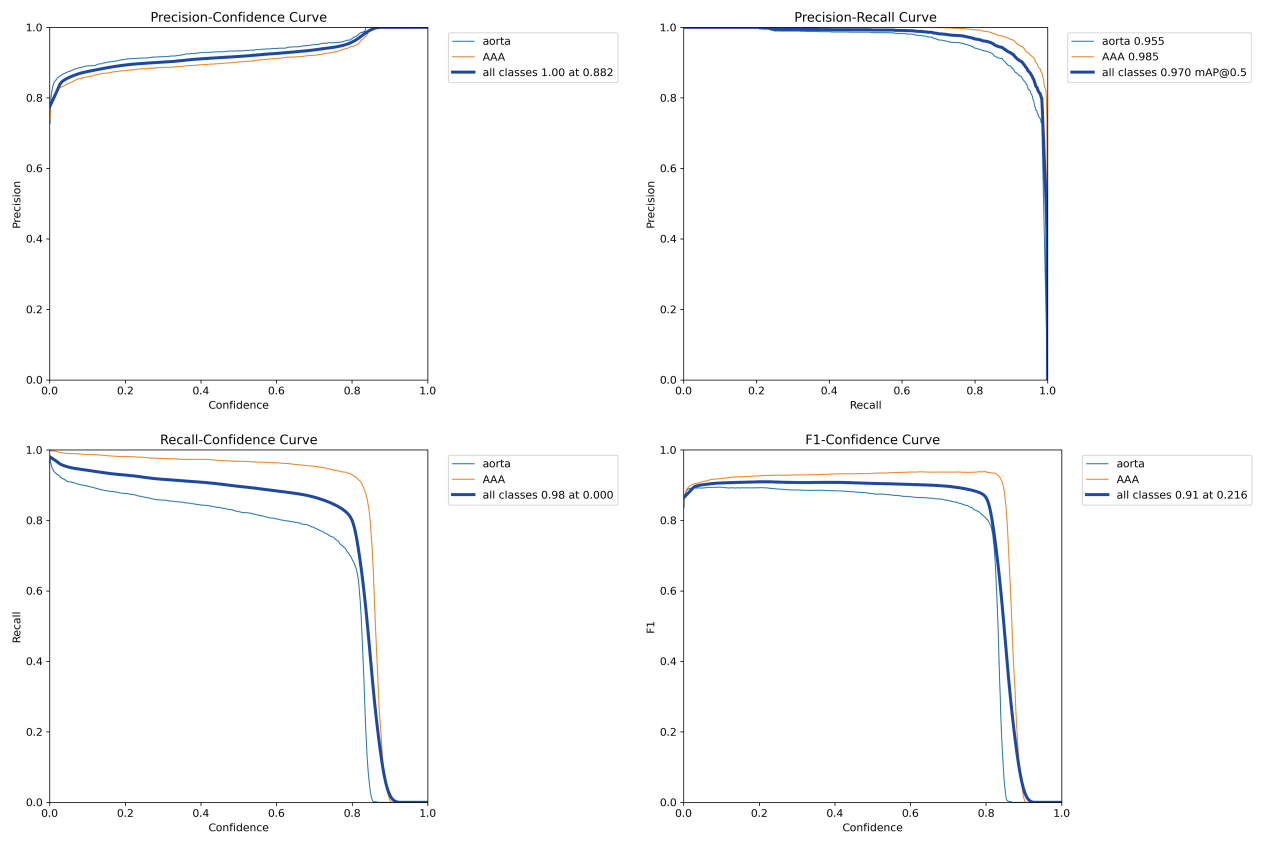


Figure 12. Confusion matrices of the YOLOv5 model for classifying normal aorta and abdominal aortic aneurysm. YOLO: You Only Look Once.


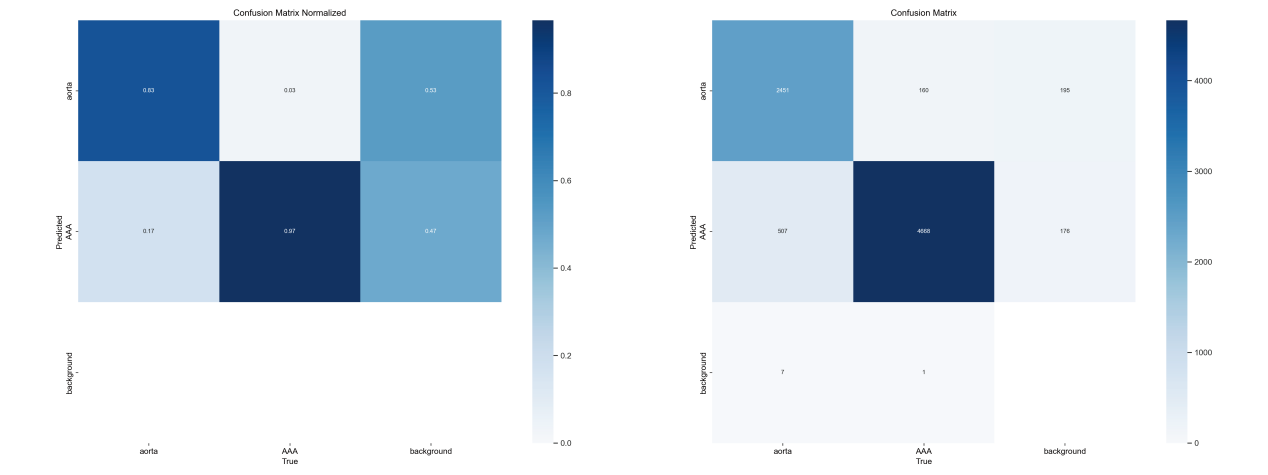


Figure 13. Training and validation losses with performance metrics of the YOLOv5 model for detecting and classifying the abdominal aorta. YOLO: You Only Look Once.


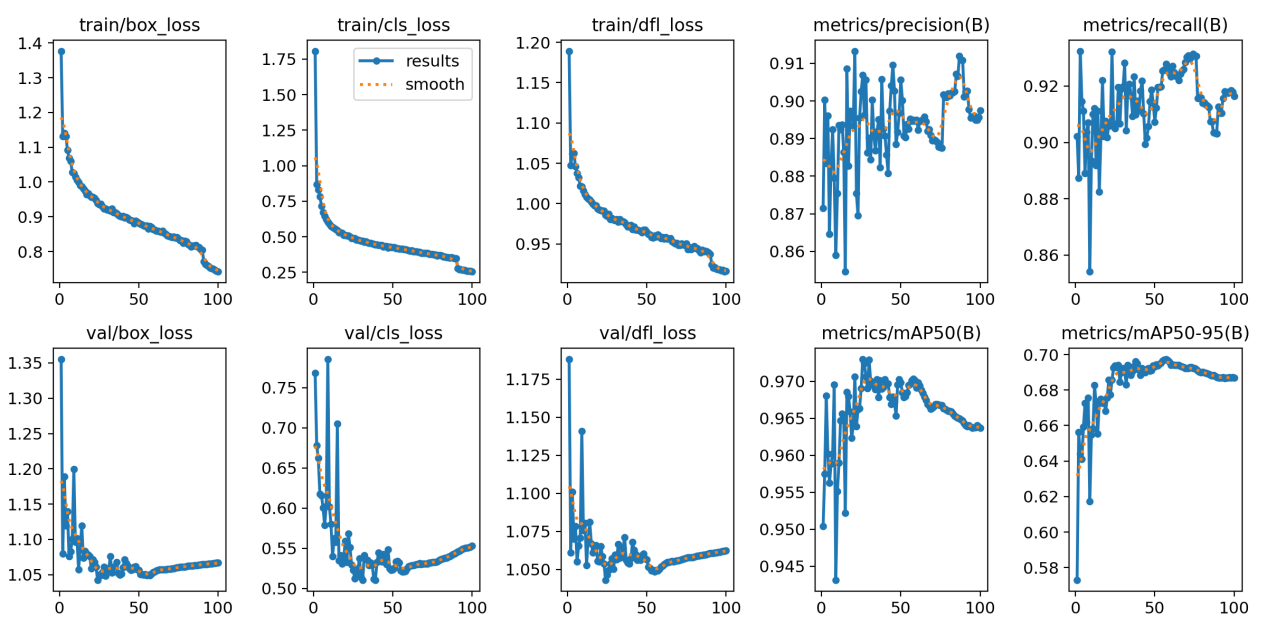


Figure 14. Training for classifying the normal aorta and abdominal aortic aneurysm of the YOLOv8 model on CTA images. YOLO: You Only Look Once; CTA: Computed tomography angiography.


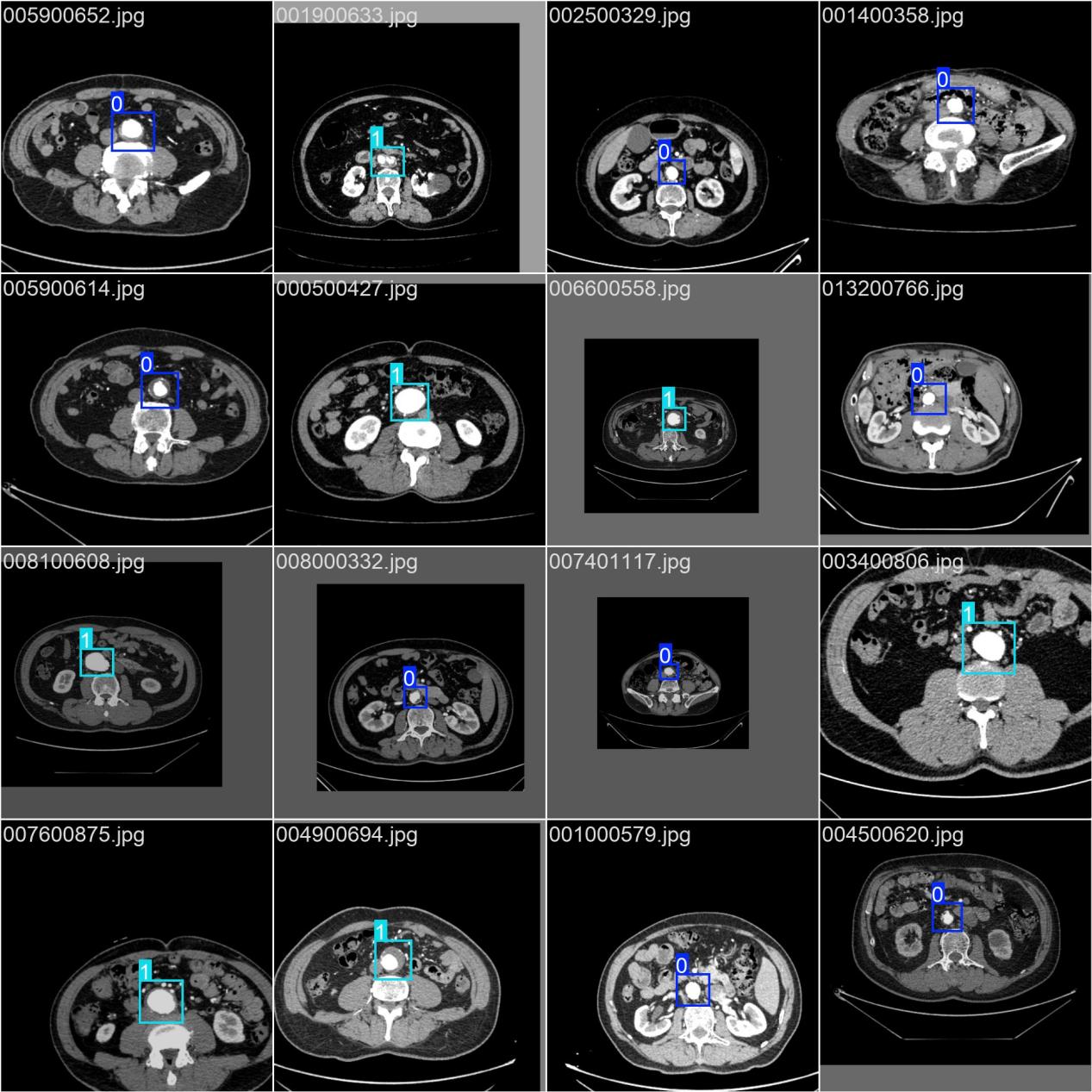


Figure15. Validation for classifying the normal aorta and abdominal aortic aneurysm of the YOLOv8 model on CTA images. YOLO: You Only Look Once; CTA: Computed tomography angiography.


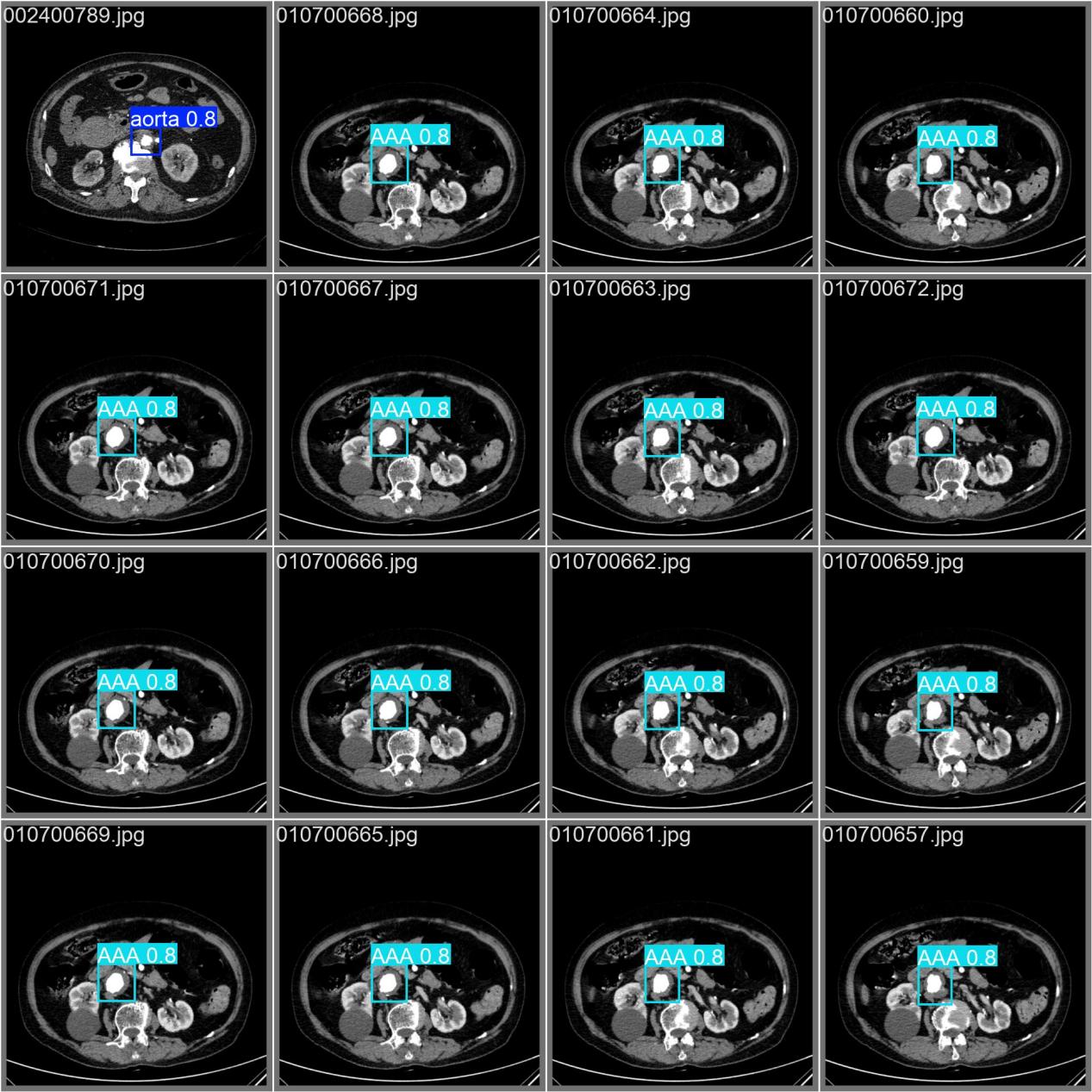


Figure 16. Performance curves of the YOLOv8 model for detecting and classifying the abdominal aorta. YOLO: You Only Look Once.


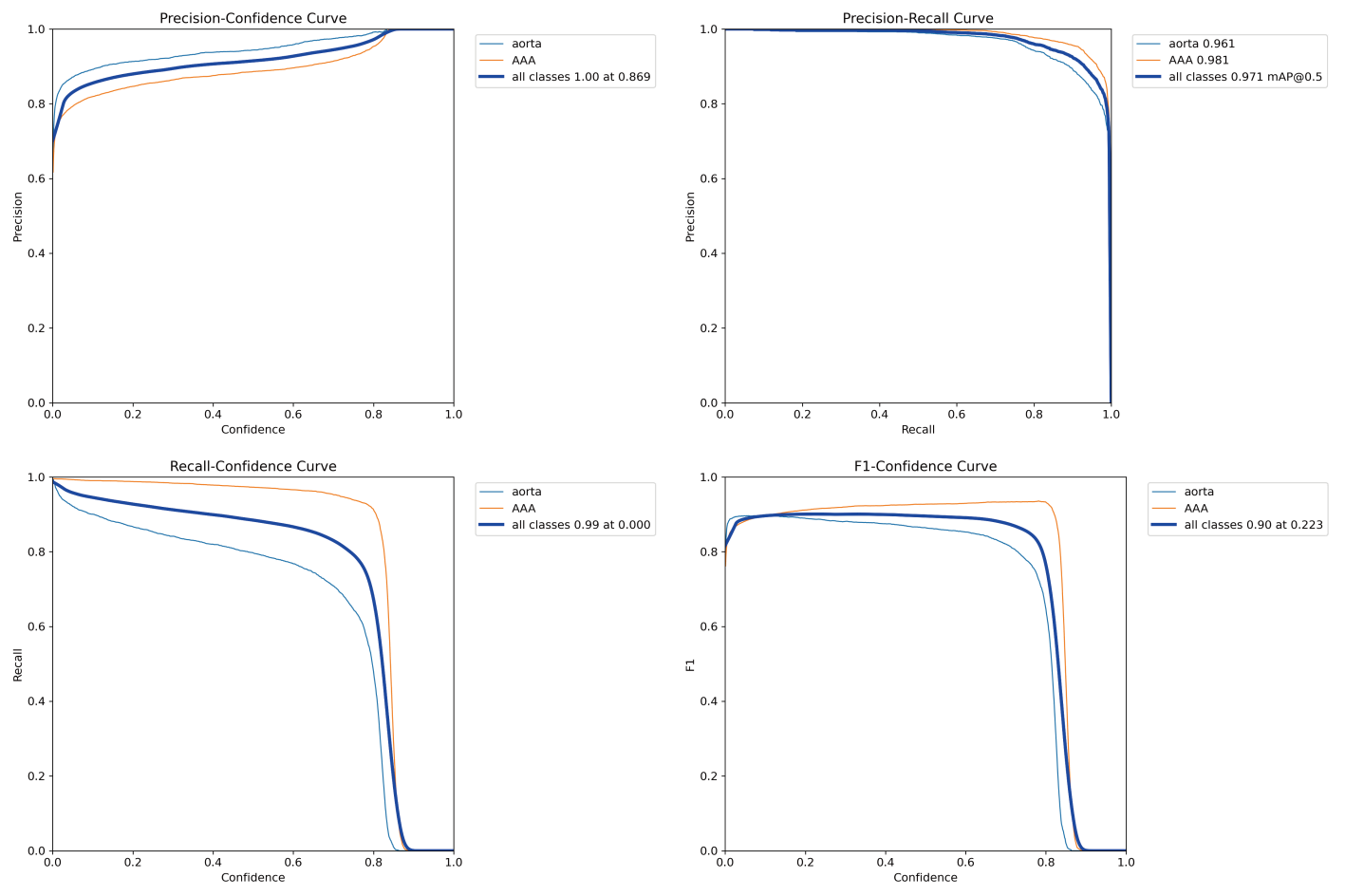


Figure 17. Confusion matrices of the YOLOv8 model for classifying normal aorta and abdominal aortic aneurysm. YOLO: You Only Look Once.


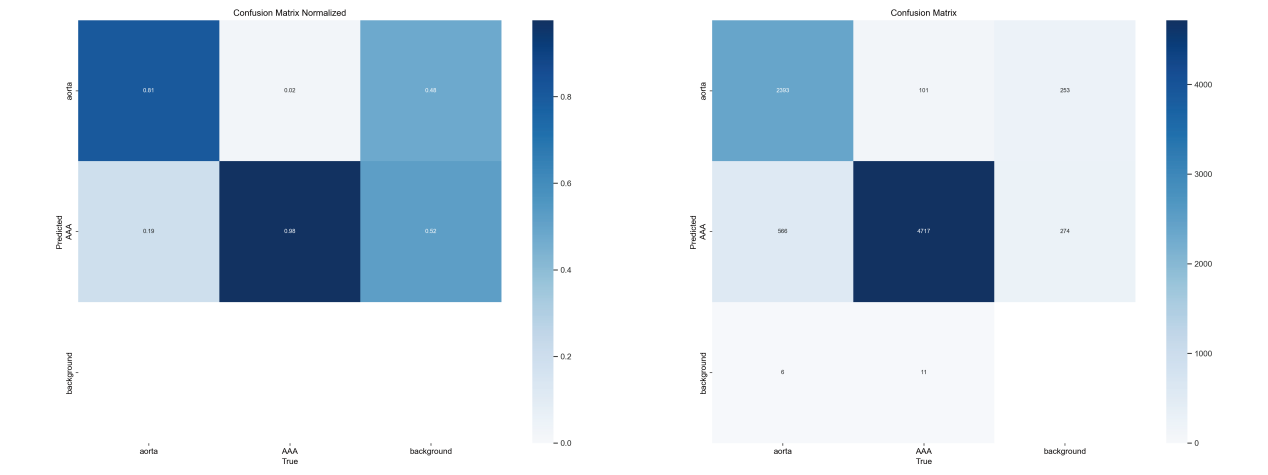


Figure 18. Training and validation losses with performance metrics of the YOLOv8 model for detecting and classifying the abdominal aorta. YOLO: You Only Look Once.


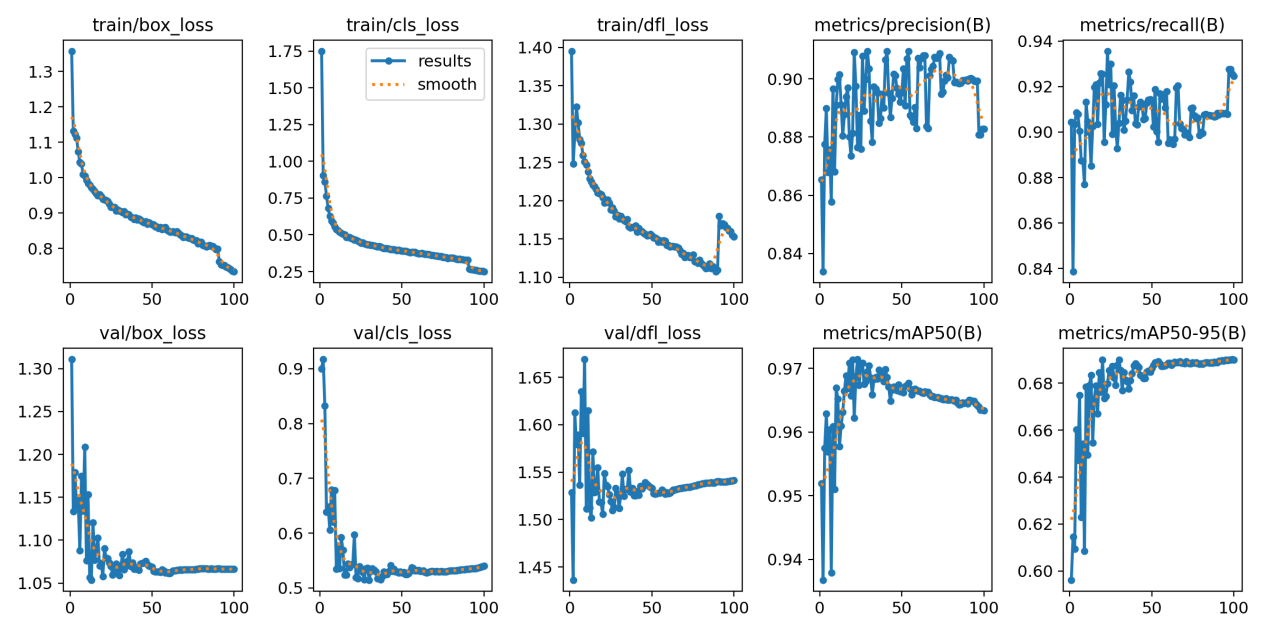


Figure 19. Training for classifying the normal aorta and abdominal aortic aneurysm of the YOLOv10 model on CTA images. YOLO: You Only Look Once; CTA: Computed tomography angiography.


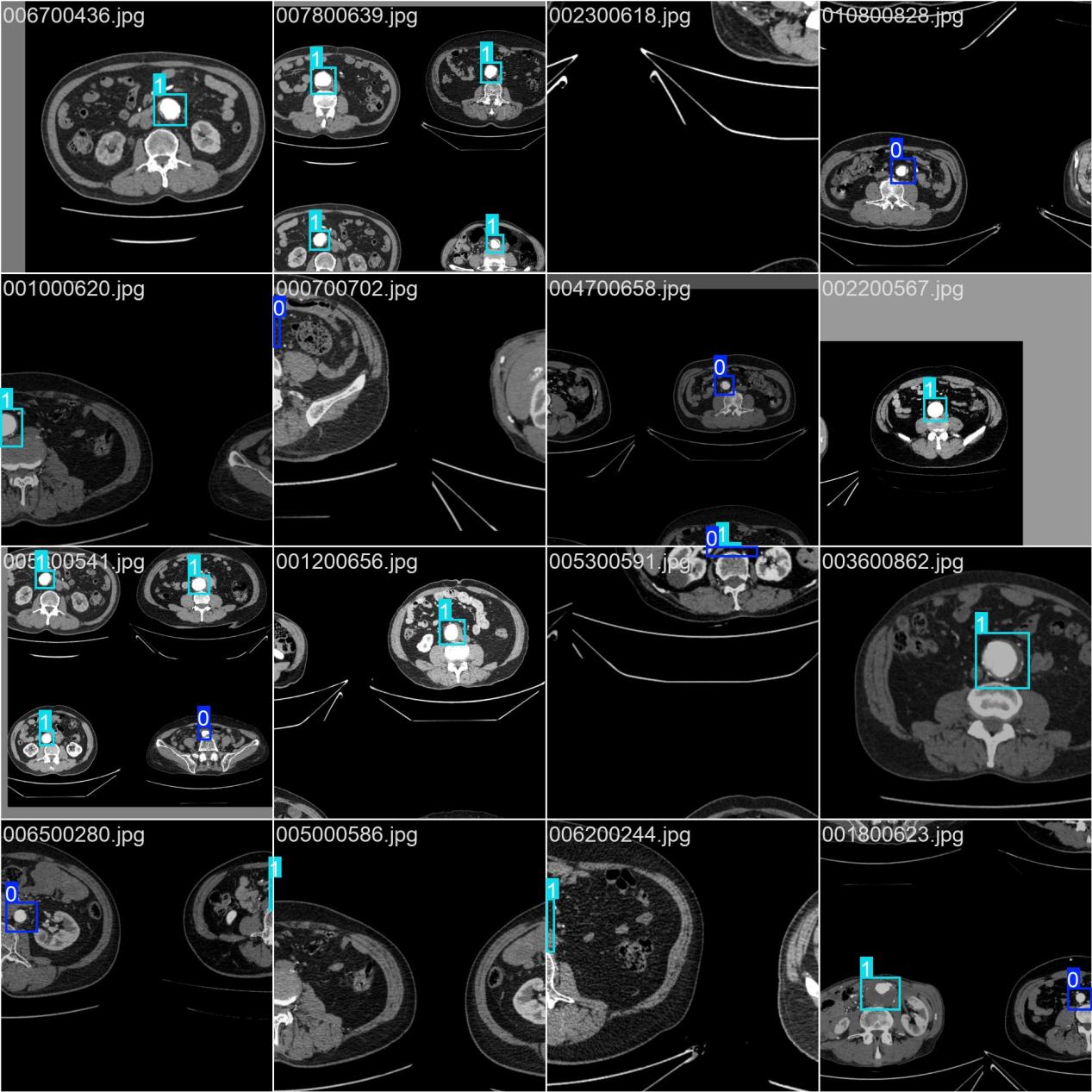


Figure 20. Validation for classifying the normal aorta and abdominal aortic aneurysm of the YOLOv10 model on CTA images. YOLO: You Only Look Once; CTA: Computed tomography angiography.


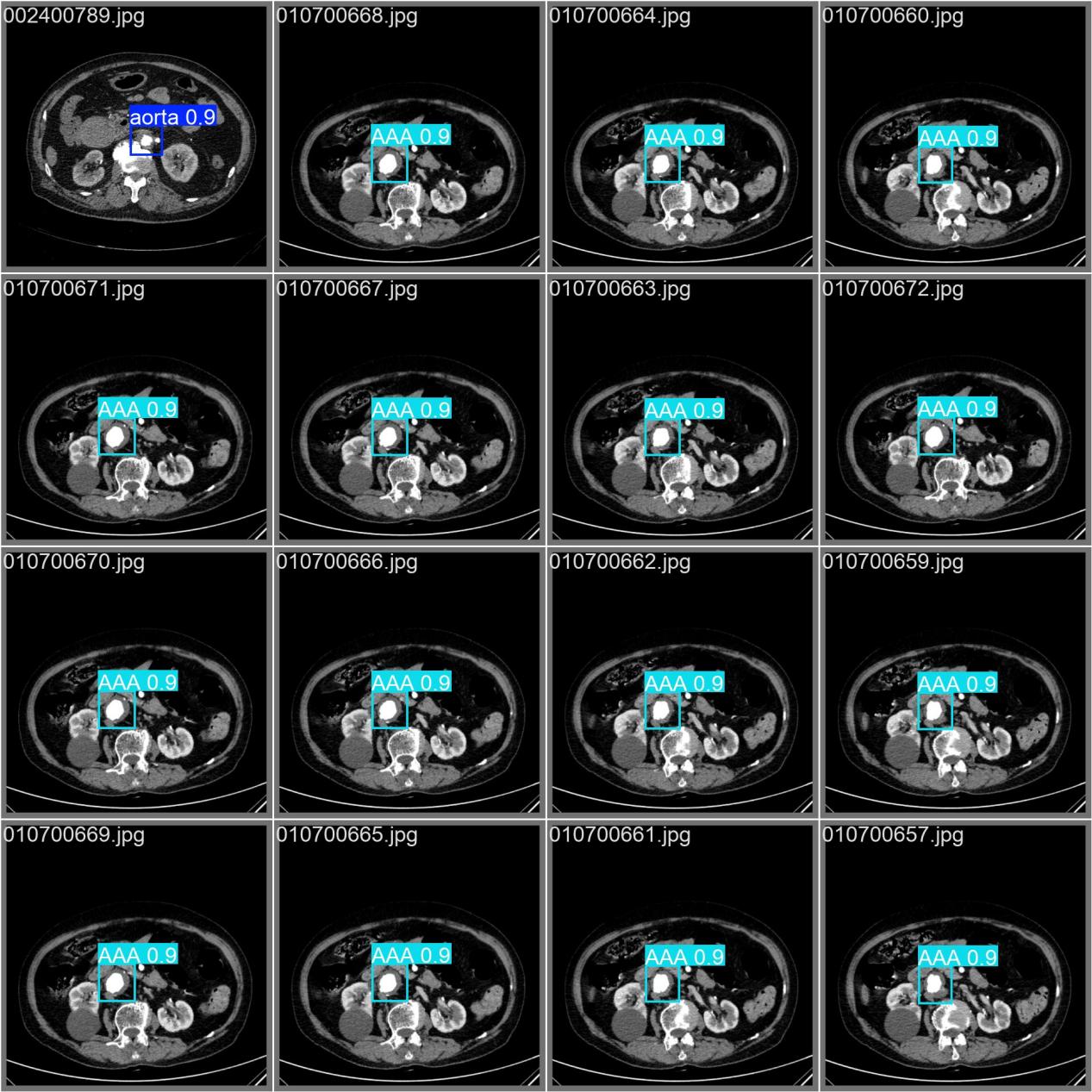


Figure 21. Performance curves of the YOLOv10 model for detecting and classifying the abdominal aorta. YOLO: You Only Look Once.


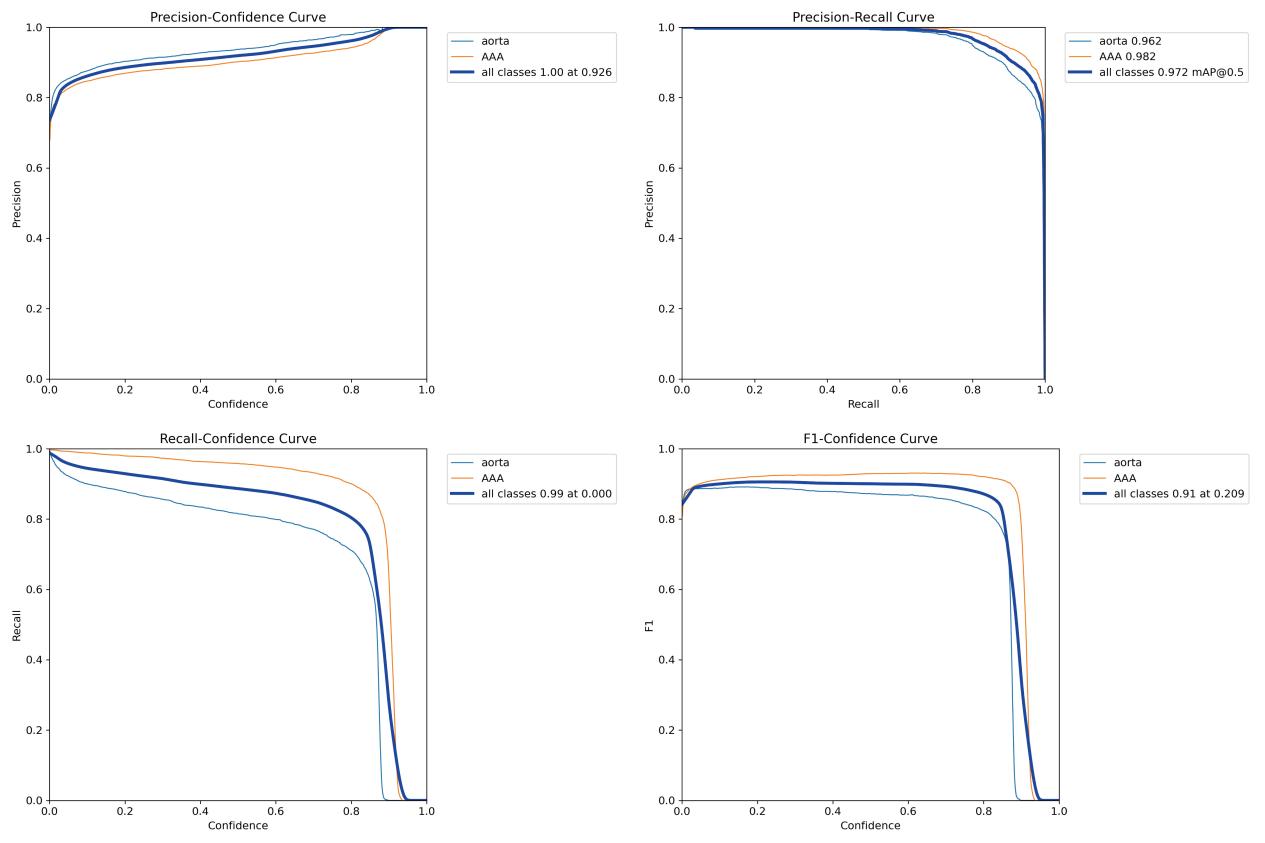


Figure 22. Confusion matrices of the YOLOv10 model for classifying normal aorta and abdominal aortic aneurysm. YOLO: You Only Look Once.


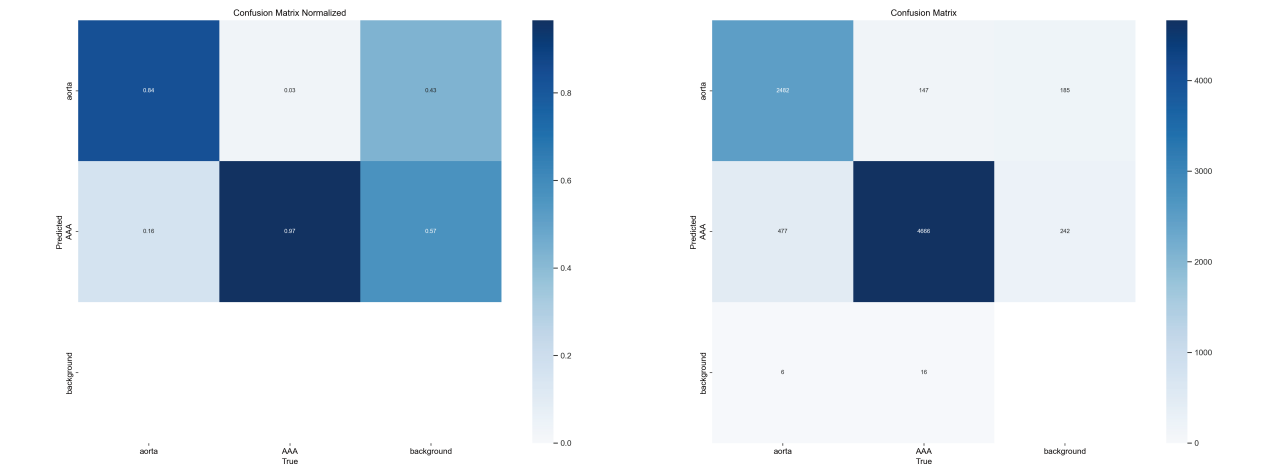


Figure 23. Training and validation losses with performance metrics of the YOLOv10 model for detecting and classifying the abdominal aorta. YOLO: You Only Look Once.


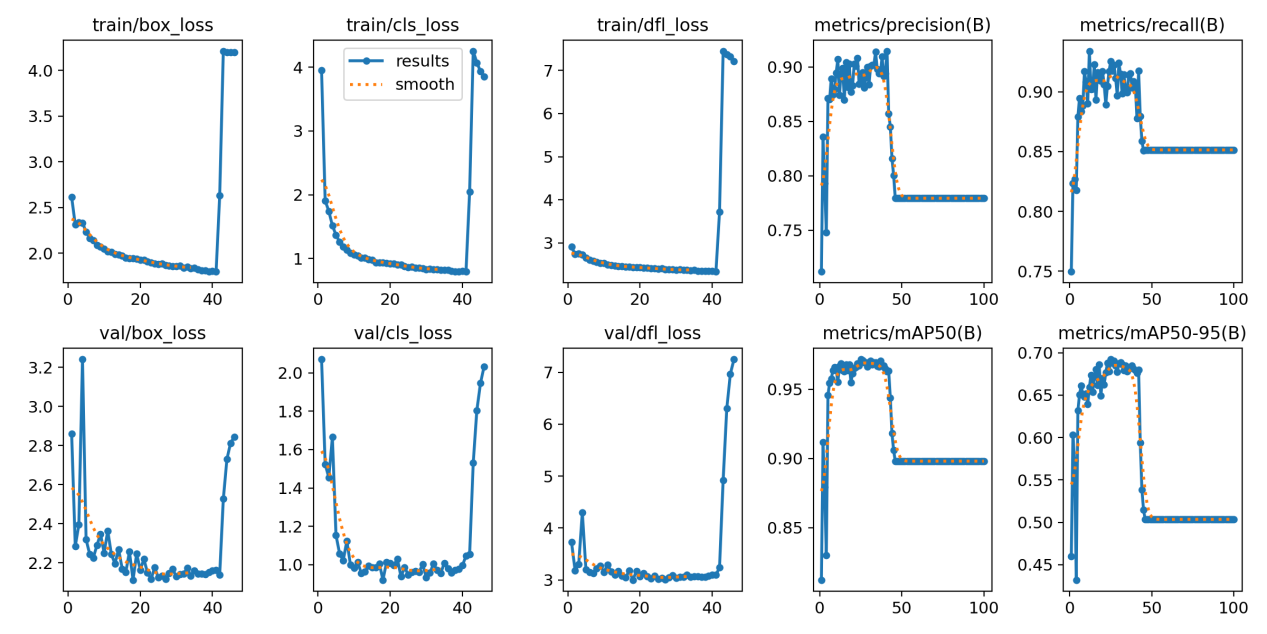


Figure 24. Training and validation losses of the feature fusion model based on the VGG16 backbone.


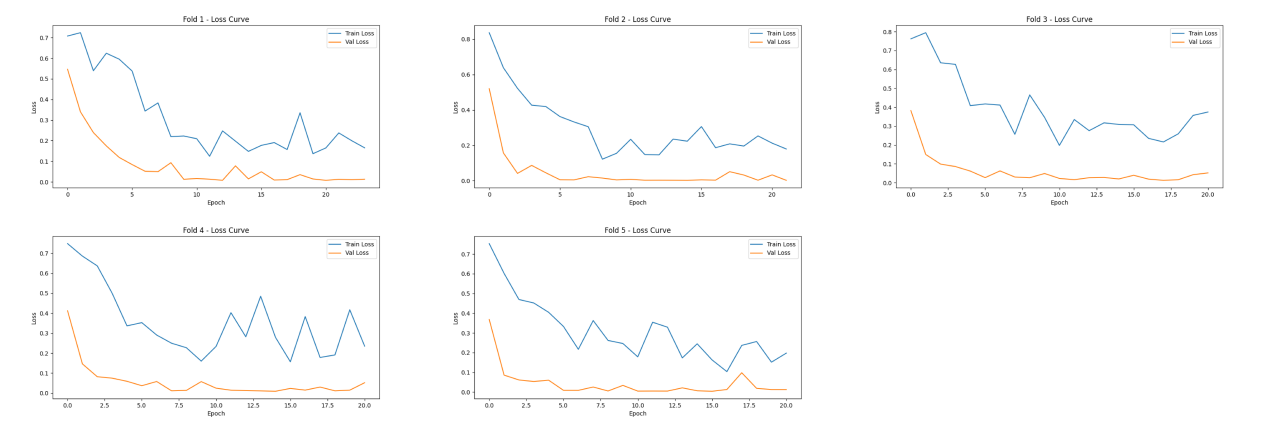


Figure 25. Confusion matrices for the feature fusion model based on the VGG16 backbone.


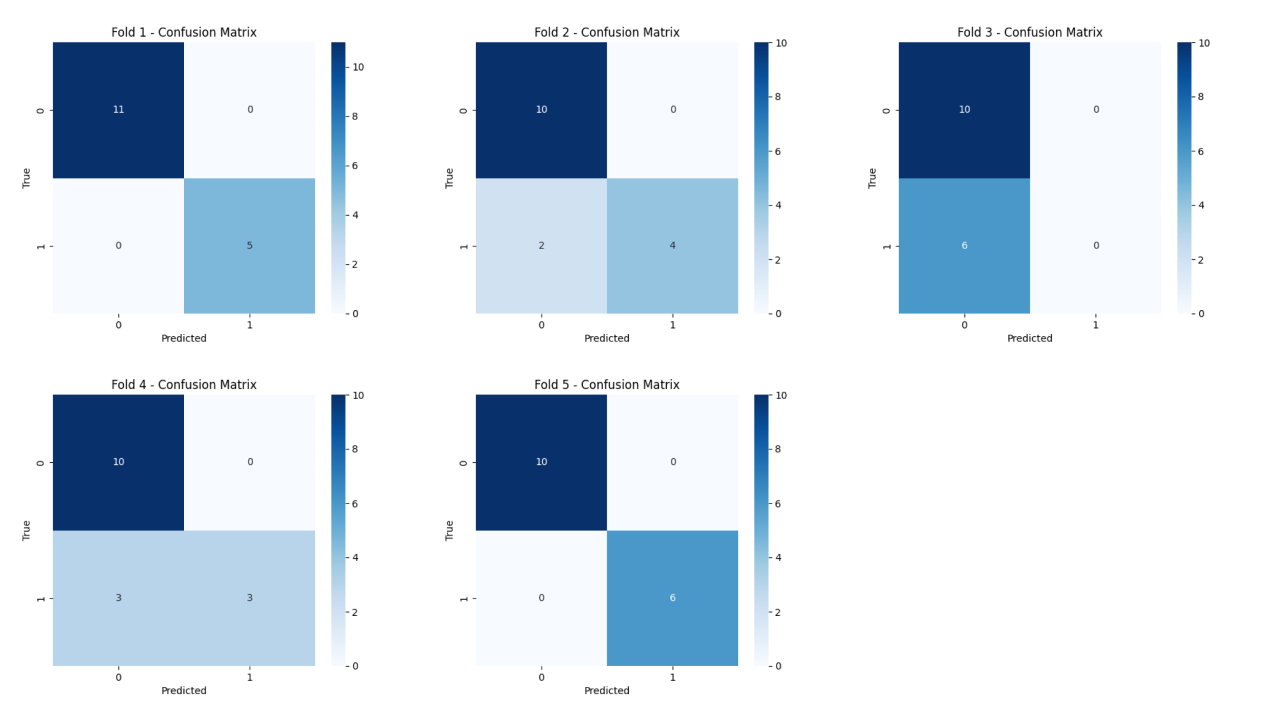


Figure 26. Training and validation losses of the feature fusion model based on the ResNet18 backbone.


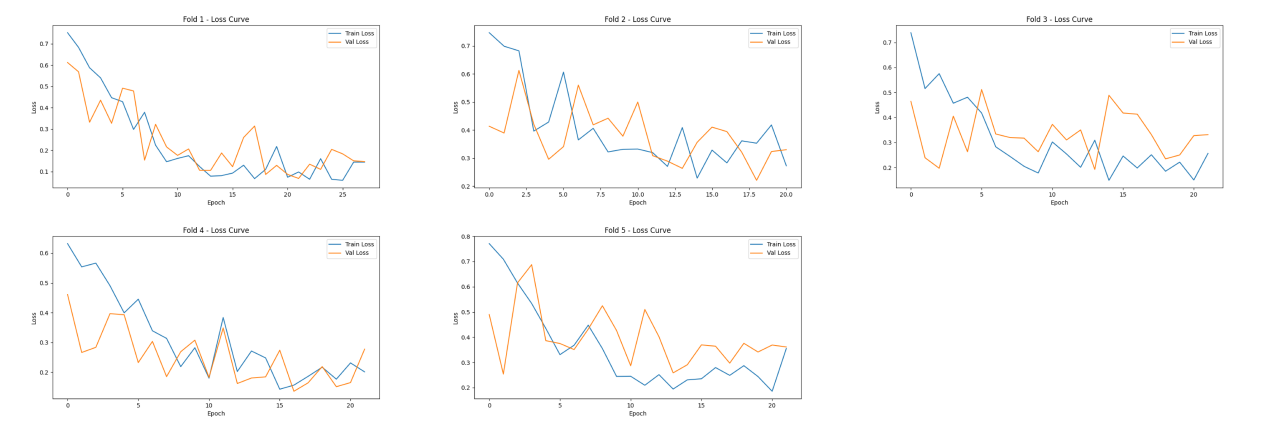


Figure 27. Confusion matrices for the feature fusion model based on the ResNet18 backbone.


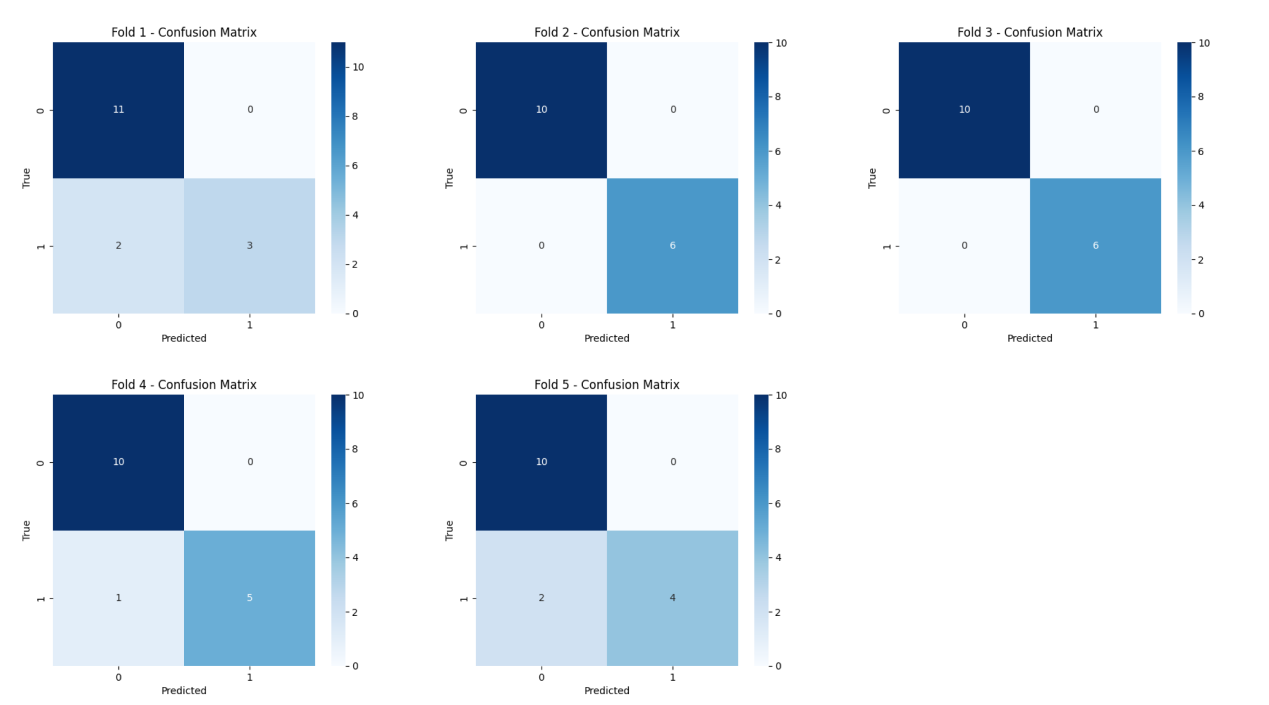


Figure 28. Training and validation losses of the feature fusion model based on the ResNet50 backbone.


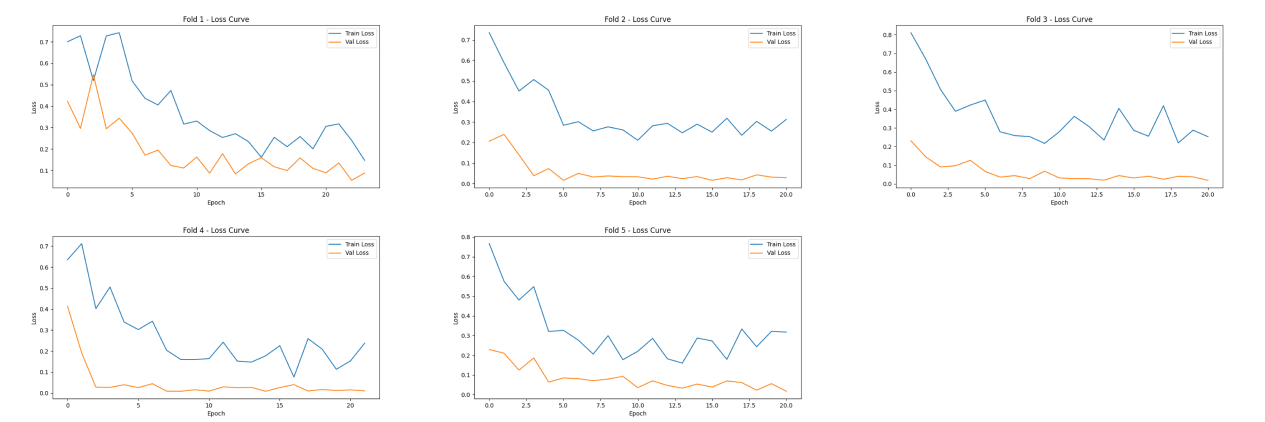


Figure 29. Confusion matrices for the feature fusion model based on the ResNet50 backbone.


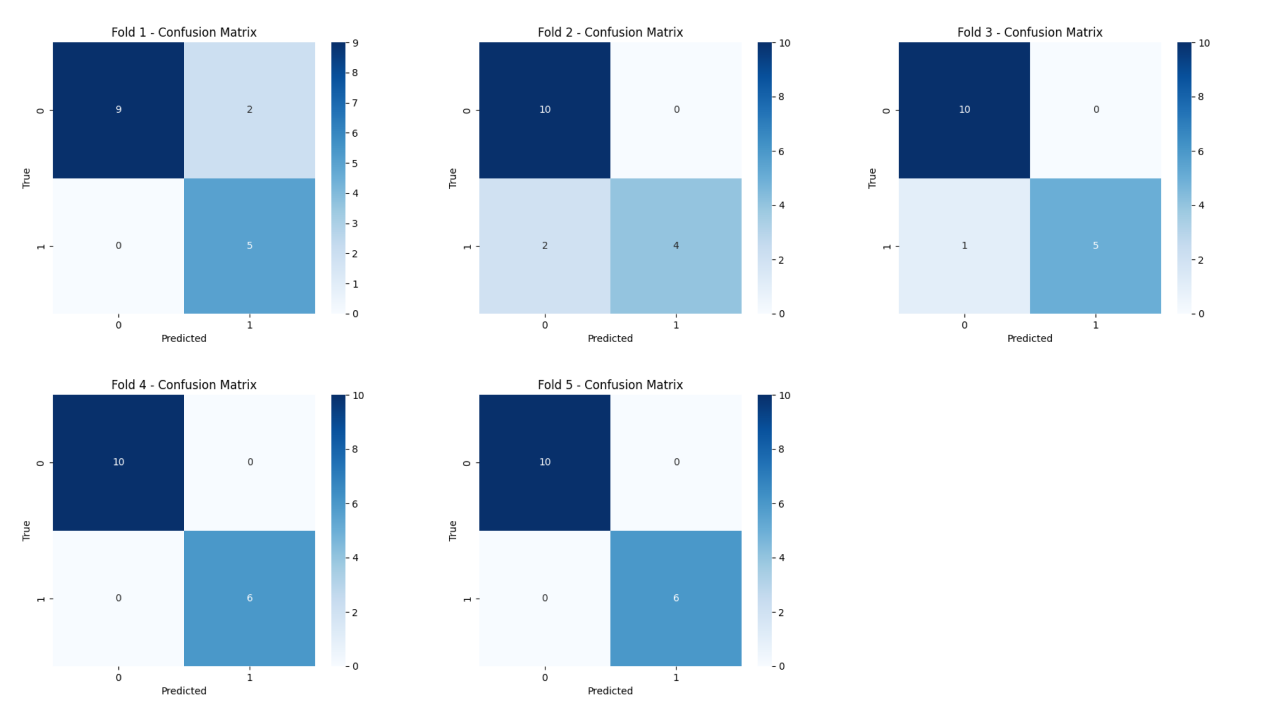


Figure 30. Training and validation losses of the feature fusion model based on the ResNet101 backbone.


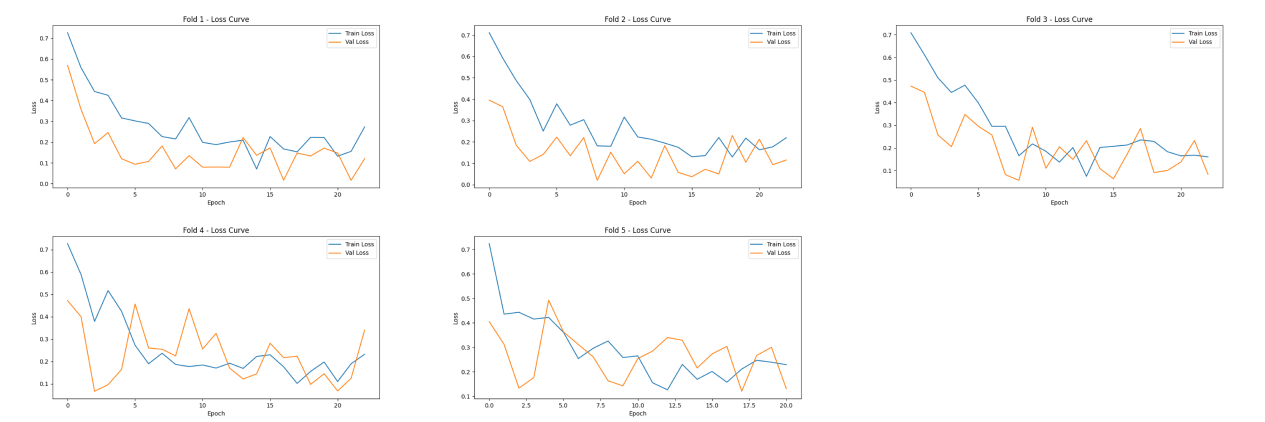


Figure 31. Confusion matrices for the feature fusion model based on the ResNet101 backbone.


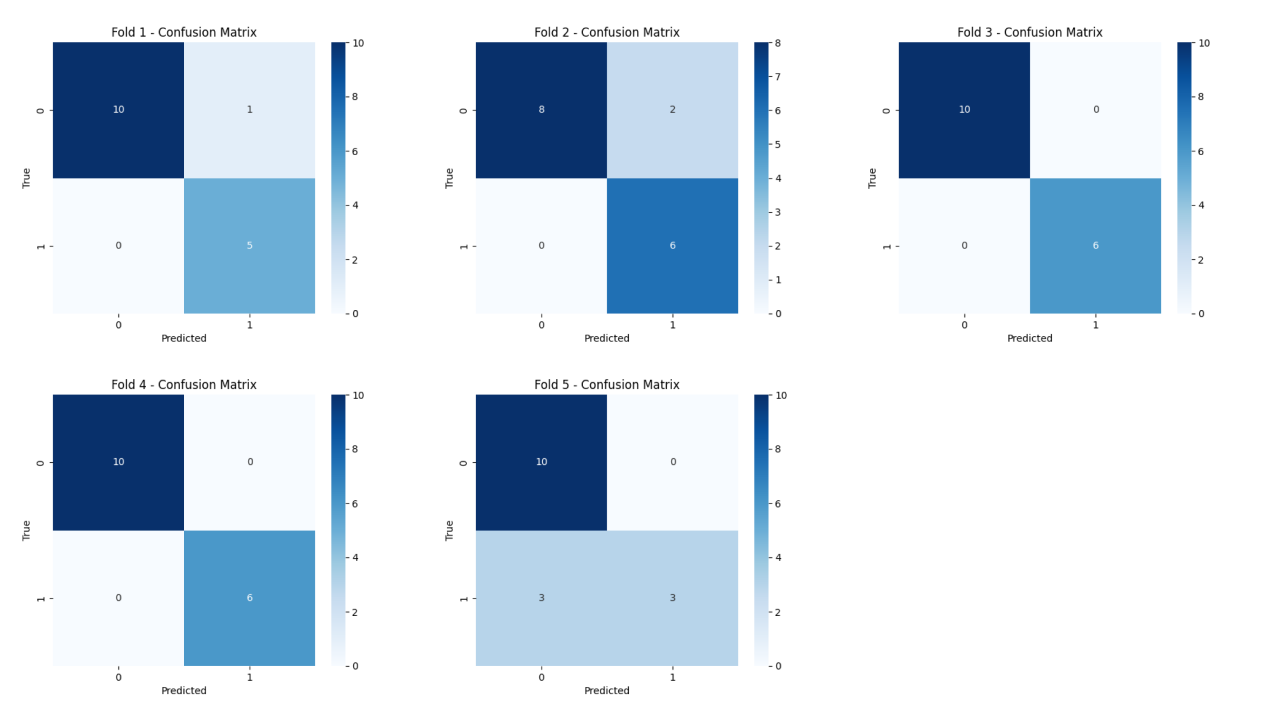


Figure 32. Training and validation losses of the feature fusion model based on the DenseNet121 backbone.


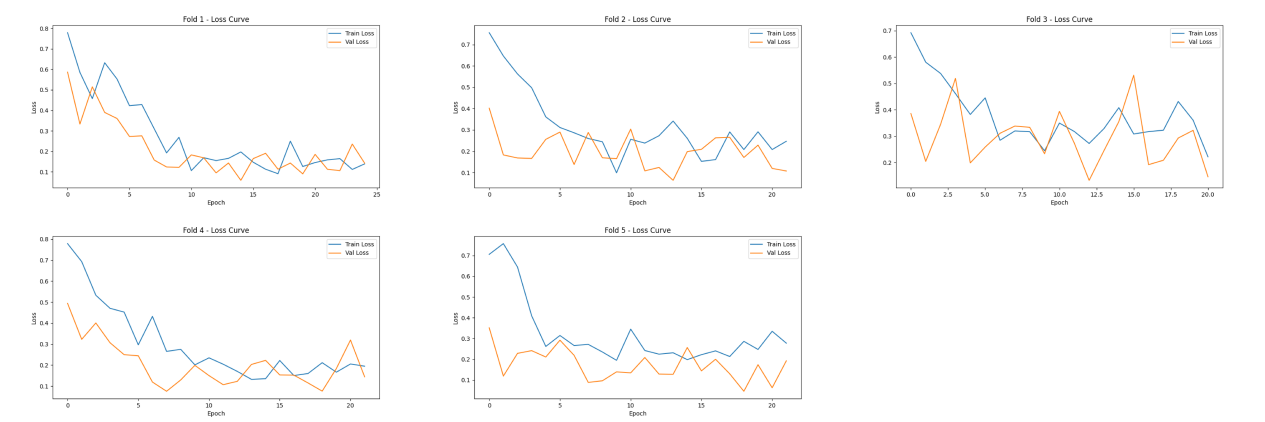


Figure 33. Confusion matrices for the feature fusion model based on the DenseNet121 backbone.


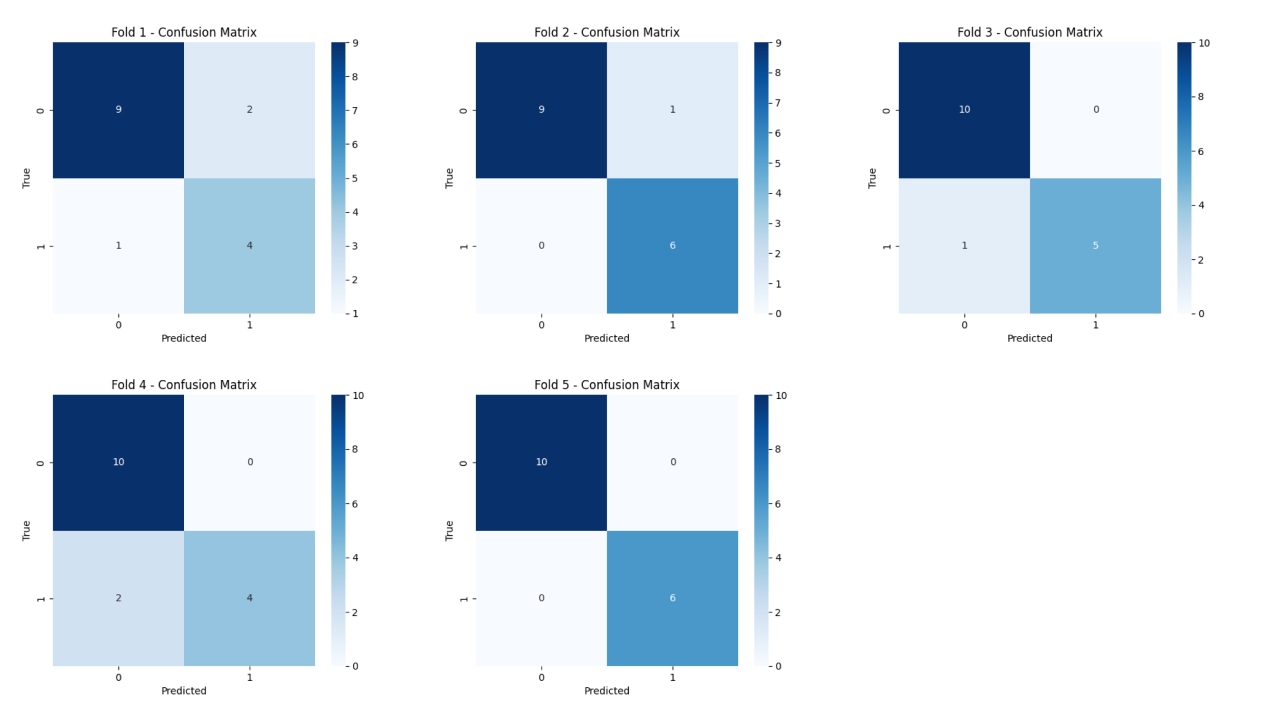


Figure 34. Training and validation losses of the feature fusion model based on the ViT-B/16 backbone.


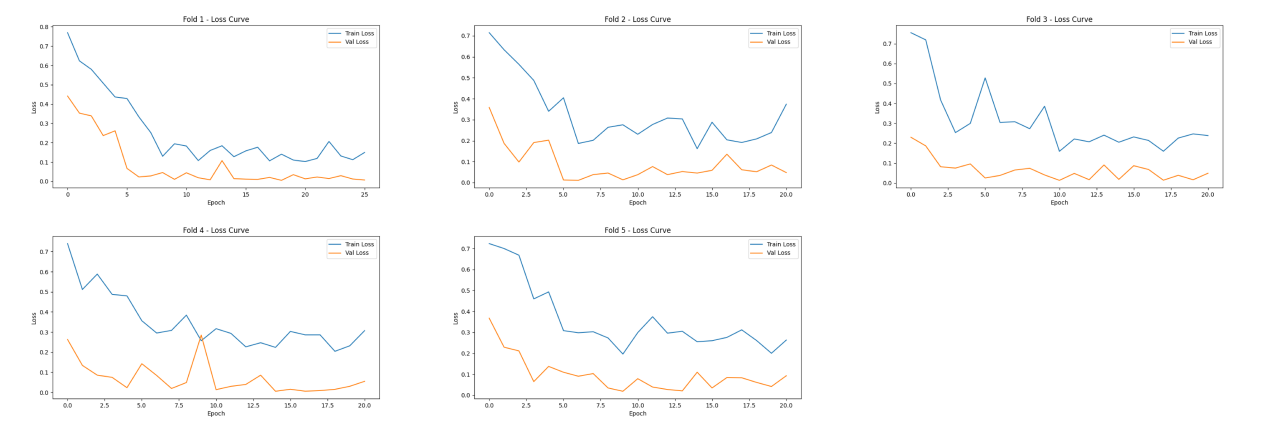


Figure 35. Confusion matrices for the feature fusion model based on the ViT-B/16 backbone.


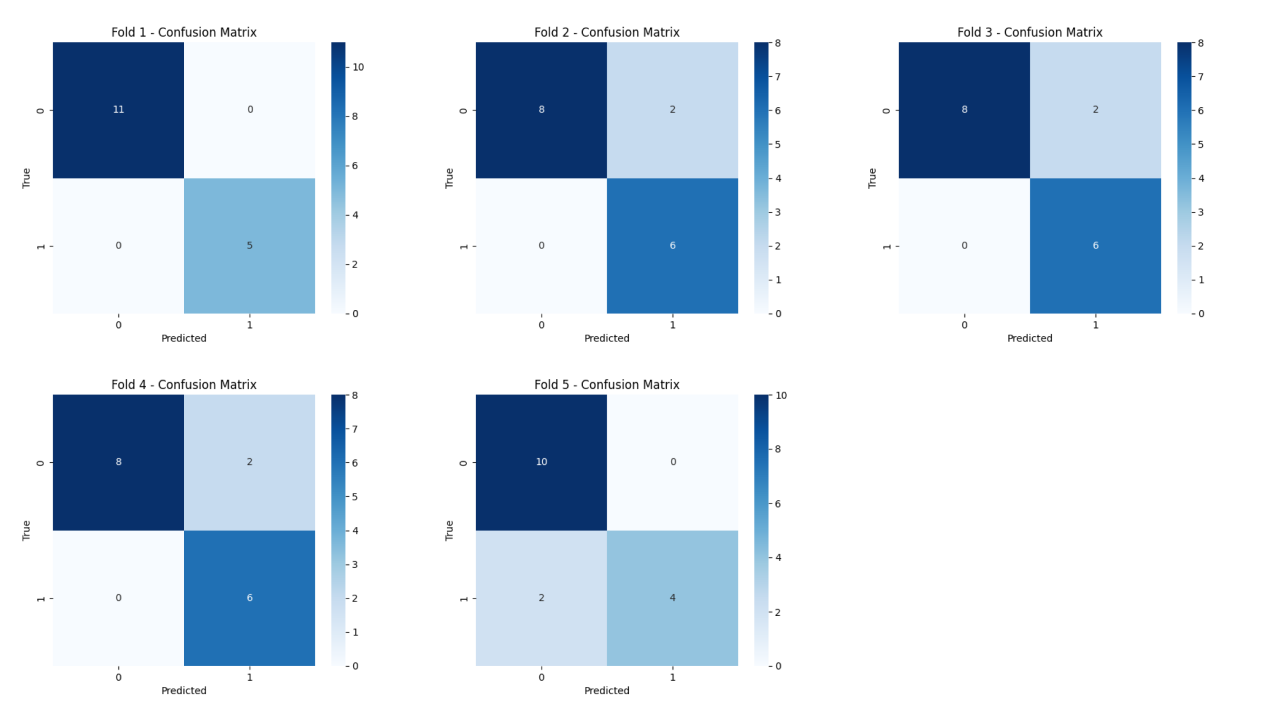


Figure 36. Training and validation losses of the feature fusion model based on the MedViT backbone.


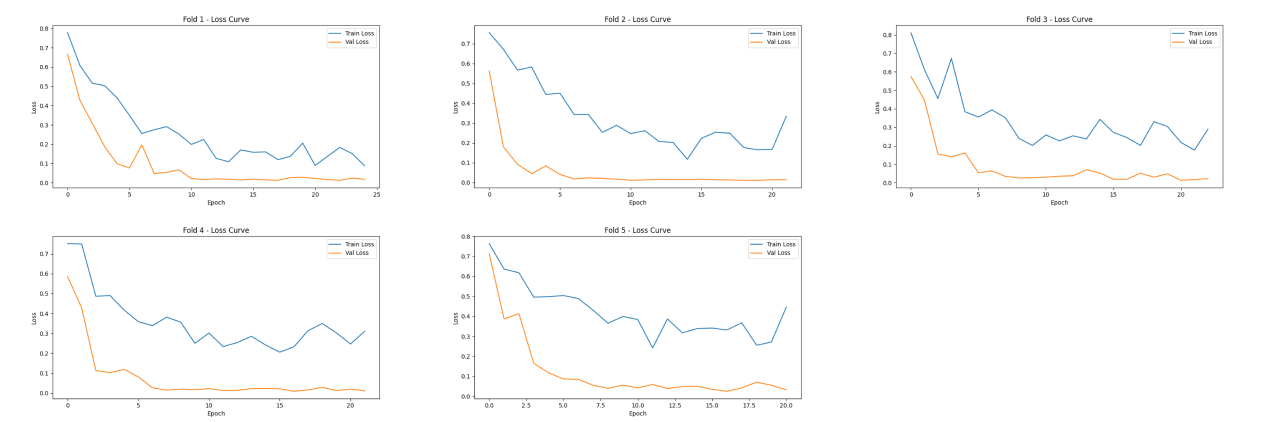


Figure 37. Confusion matrices for the feature fusion model based on the MedViT backbone.


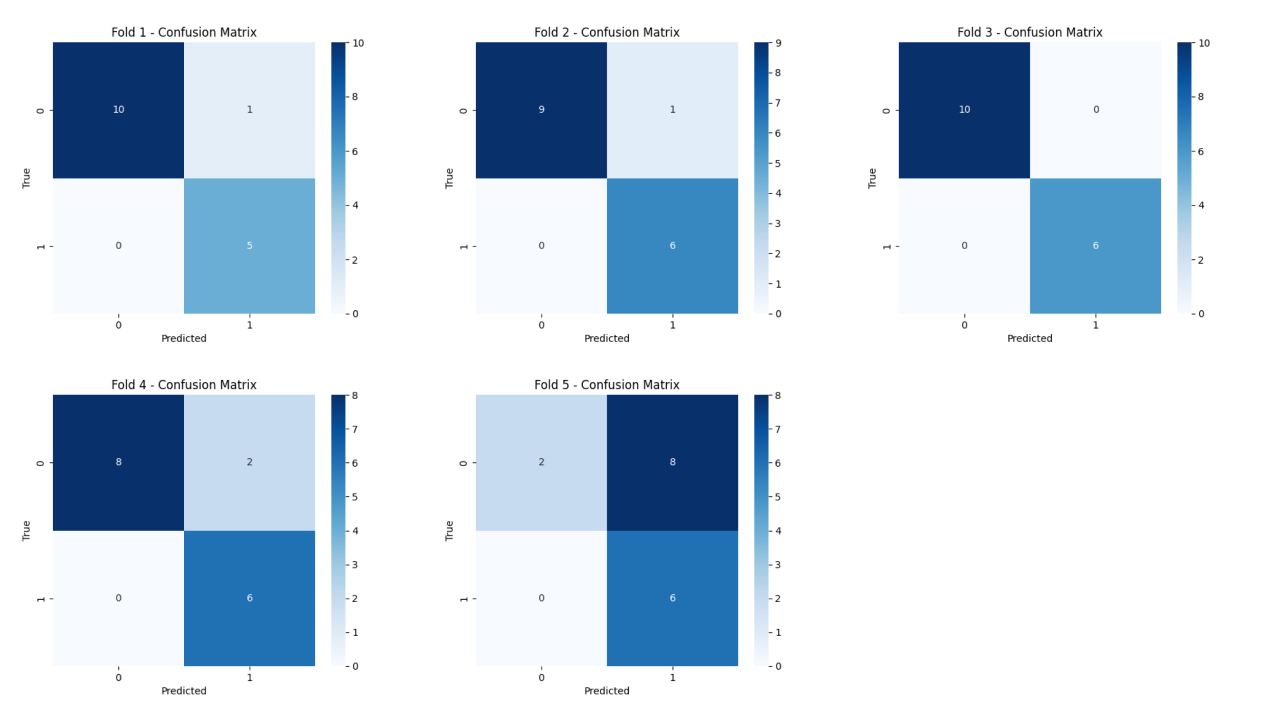

Supplement: Supplementary file 1 [file Table1.docx]
