# Supplementary material for: Predicting the growth of asymptomatic small abdominal aortic aneurysms (AAA) based on deep learning
Source: Front Physiol. 2026 Jan 27;16:1704428. doi: 10.3389/fphys.2025.1704428 (PMC12886037; doi:10.3389/fphys.2025.1704428)
Supplement: Supplementary file 2 [file Table2.docx]

Supplements.

Table 1. Summary of experimental data on CTA Images.

| Types | Training set | Validation set | Test set | Total |
| --- | --- | --- | --- | --- |
| CTA images containing the abdominal aorta | 25,165 | 3,145 | 3,147 | 31,457 |
| CTA images containing no abdominal aorta | 120,667 | 15,083 | 15,084 | 150,834 |

Table 2. CTA images for detecting and classifying the abdominal aorta.

| Types | Train set | Valid set | Test set | Total |
| --- | --- | --- | --- | --- |
| CTA images of abdominal aorta | 15,704 | 7,794 | 7,964 | 31,457 |

Formula.

1. $Recall=\frac{TP}{TP+FN}$
2. $Precision=\frac{TP}{TP+FP}$
3. $F1 score=2\times\frac{Recall\times Precision}{Recall+Precision}$
4. $Accuracy=\frac{TP+TN}{TP+TN+FP+FN}$

TN: True Negative; FP: False Positive; FN: False Negative; TP: True Positive.
